# Mapping the characterization landscape of *Colocasia esculenta* (L.) in Africa: gaps, trends, and priorities - a systematic review with meta-analysis

**DOI:** 10.3389/fpls.2026.1930197

**Published:** 2026-09-21

**Authors:** Riwan Jaures Uriel Kouke, Beket Severin Bonny, Nwonumara Godwin Nkwuda, Nwanchor Kelechi, Corneille Ahanhanzo, Kouamé Kevin Koffi

**Affiliations:** 1 Laboratory of Functional Genomics and Genetic Improvement, Faculty of Natural Sciences University Nangui Abrogoua, Abidjan, Côte d'Ivoire; 2 Central Laboratory of Plant Biotechnology and Plant Breeding, Department of Genetics and Biotechnology, Faculty of Science and Technique, University of Abomey-Calavi, Abomey-Calavi, Cotonou, Benin; 3 Department of Applied Biology, Faculty of Science, Ebonyi State University, Abakaliki, Nigeria; 4 Staff Fellow, Orphan Project, UFR Sciences de la Nature, Nangui Abrogoua University, Abidjan, Côte d'Ivoire; 5 Department of Crop Production and Landscape Management, Faculty of Agriculture and Natural Resources Management, Ebonyi State University, Abakaliki, Nigeria

**Keywords:** expected heterozygosity, genetic diversity, orphan crop, polymorphism information content, population structure, PRISMA

## Abstract

Taro (*Colocasia esculenta* (L.) Schott) is a vegetatively propagated root crop of major importance for food security in Africa. Despite this, no continental synthesis has integrated its agromorphological, biochemical, and molecular characterization findings. This systematic review with meta-analysis aimed to (i) compile all studies characterizing *Colocasia esculenta* in Africa between 2010 and 2026, (ii) estimate pooled genetic diversity through random-effects meta-analyses, and (iii) identify key research gaps. The review followed PRISMA 2020 guidelines, with the protocol prospectively registered on the Open Science Framework (osf.io/5s9py; DOI: 10.17605/OSF.IO/5S9PY). Five electronic databases were searched for English-language articles published between January 2010 and April 2026. Screening was performed by two independent reviewers using Rayyan in blind mode (Cohen’s kappa = 0.84). Methodological quality was assessed using an *ad hoc* scoring tool adapted from the Joanna Briggs Institute tools. Random-effects meta-analyses were performed on expected heterozygosity (He), polymorphism information content (PIC), and number of alleles per locus (Na), with marker-stratified analyses (SSR vs SNP/GBS) as primary analyses and meta-regression for four moderators. Of 222 records identified, 64 studies were included (44 original articles and 20 reviews). The geographic distribution was uneven, with Nigeria, Kenya, South Africa, and Ethiopia accounting for 86% of original studies, while Central and North Africa were essentially unrepresented. The methodological landscape shifted toward SSR (58.8%) and SNP/GBS (29.4%) markers recently. Pooled estimates were He = 0.40 (95% CI: 0.32-0.48; 95% PI: 0.08-0.72; k = 12), PIC = 0.46 (95% CI: 0.38-0.55; 95% PI: 0.18-0.75; k = 10), and Na = 3.72 (95% CI: 3.13-4.30; 95% PI: 1.63-5.81; k = 10). The wide prediction intervals should be considered the primary reference range for future studies, and marker-stratified estimates are preferred over overall pooled values for cross-study comparisons. Between-study heterogeneity was high (I^2^ > 89%) and the English-only restriction may have led to the underrepresentation of francophone African studies. African taro harbors moderate genetic diversity, yet the literature is geographically skewed, biochemical characterization is underrepresented, and no studies integrate all three dimensions. Future research should prioritize collection in underrepresented regions, multi-dimensional characterization, SNP-based genotyping, and core collection development.**Systematic review registration:** https://osf.io/5s9py/, identifier 10.17605/OSF.IO/5S9PY.

## 1 Introduction

Among the root and tuber crops cultivated across the humid tropics, taro (*Colocasia esculenta* (L.) Schott) occupies a paradoxical position. It ranks as the fourteenth most consumed vegetable globally, feeds an estimated 500 million people worldwide, and sustains rural livelihoods across more than 65 countries (Rao et al., 2010; Lebot, 2009). Yet despite this widespread importance, taro has received a fraction of the research investment directed at crops of comparable economic significance such as cassava, yam, or sweet potato. The designation of taro as an “orphan” or “neglected and underutilized” crop (Padulosi et al., 2013) reflects not a lack of agronomic potential but rather a persistent gap between the crop’s actual contribution to food systems and the scientific attention it commands.

This disparity between the agricultural importance of taro and the attention it receives is particularly pronounced in Africa, where the production accounts for approximately 10 million tons annually, representing about 40% of global production, led by countries such as Nigeria, Cameroon, Ghana, and Ethiopia (FAOSTAT, 2023). Taro serves as a staple food in parts of West and Central Africa, a dietary supplement in East Africa, and a culturally significant crop in communities from the Nile Delta to the shores of Lake Victoria. The corms and leaves provide carbohydrates, dietary fiber, and essential minerals, while the crop’s tolerance of waterlogged soils, shade, and marginal conditions makes it a valuable component of climate-resilient farming systems (Chivenge et al., 2015; Oladimeji et al., 2022). In regions where drought, soil degradation, and unpredictable rainfall threaten cereal-based food security, taro offers an alternative that is both nutritionally adequate and ecologically appropriate.

The characterization of available germplasm is a prerequisite for effective conservation and utilization of plant genetic resources in breeding programs (Emmanuel et al., 2025). In plant genetic resource science, characterization serves two essential purposes: it documents the range of variation present within a crop species, and it identifies traits of potential value for breeding, agronomy, and nutrition (Bioversity International, 2010). Three complementary approaches are conventionally employed for this purpose. Agromorphological characterization, based on the systematic scoring of phenotypic descriptors, provides a rapid and low-cost assessment of visible variation in plant architecture, leaf morphology, corm attributes, and yield components. Biochemical characterization quantifies the nutritional and anti-nutritional composition of edible plant parts, including starch, protein, mineral, and oxalate contents. Molecular characterization, using DNA-based marker systems, reveals genetic variation at the genome level, independent of environmental effects and developmental stage. Each approach captures a different dimension of diversity, and their combined use provides the most comprehensive portrait of a crop’s genetic resources (Mohammadi and Prasanna, 2003).

Over the past two decades, the tools available for taro characterization have undergone substantial evolution. Early studies relied predominantly on morphological descriptors standardized by the International Plant Genetic Resources Institute (IPGRI, 1999), complemented by isozyme analyses. The advent of PCR-based molecular markers in the late 1990s and early 2000s introduced new possibilities for assessing genetic relationships, with random amplified polymorphic DNA (RAPD) and amplified fragment length polymorphism (AFLP) markers providing the first genome-wide views of taro diversity (Kreike et al., 2004). The subsequent development of simple sequence repeat (SSR) markers specifically designed for *Colocasia* (Mace and Godwin, 2002) marked a turning point, enabling codominant genotyping with high reproducibility and transferability across populations. More recently, the rapid decline in sequencing costs has opened the door to single nucleotide polymorphism (SNP) genotyping through platforms such as genotyping-by-sequencing (GBS) and DArTseq, offering unprecedented marker densities for population genomic analyses (Helmkampf et al., 2018).

This methodological progression has generated a growing body of characterization data for taro across Africa; however, the available literature remains fragmented across studies and regions. Individual studies have characterized local or national germplasm collections using one or occasionally two of the three approaches, but no synthesis has attempted to integrate their findings at the continental scale. The absence of such a synthesis means that fundamental questions remain unanswered. How much genetic diversity does African taro harbor? Which molecular markers have proven most informative for assessing taro diversity, and how has the choice of marker system influenced the reported diversity estimates? Are there geographic regions of the continent where taro diversity has been well documented and others where it remains essentially unknown? What traits have been consistently identified as the most discriminating in agromorphological evaluations, and do the patterns of morphological variation align with those revealed by molecular analysis?

Systematic reviews with meta-analysis offer a rigorous framework for addressing questions of this nature. By applying transparent and reproducible search, screening, and analytical protocols, systematic reviews minimize the biases inherent in traditional narrative reviews and enable the quantitative pooling of results across studies (Page et al., 2021). In crop genetics, meta-analytical approaches have been successfully applied to assess temporal trends in cultivar diversity (Van de Wouw et al., 2010), to synthesize genetic diversity estimates across populations of parasitic organisms (Mwesigwa et al., 2024), and to evaluate the genetic structure of crop germplasm collections at regional scales (Chikasha et al., 2026). To date, however, no systematic review with meta-analysis has been conducted for taro, whether globally or for a specific geographic region.

The present study was designed to fill this gap. Its objectives are threefold. First, it aims to provide a comprehensive and systematic inventory of the agromorphological, biochemical, and molecular characterization studies conducted on *Colocasia esculenta* in Africa between 2010 and 2026, documenting the temporal, geographic, and methodological trends in this body of literature. Second, it seeks to quantify the overall genetic diversity of African taro through a random-effects meta-analysis of molecular diversity metrics, specifically expected heterozygosity (He), polymorphism information content (PIC), and number of alleles per locus (Na) by pooling estimates across studies while accounting for between-study heterogeneity. Third, it aims to identify critical gaps in the current knowledge base and to formulate targeted recommendations for future research, breeding, and conservation efforts.

By assembling and critically evaluating the available evidence within the structured framework of the PRISMA 2020 guidelines (Page et al., 2021), this review aspires to provide a reference point for researchers, breeders, and policy-makers working on taro in Africa. The protocol was prospectively registered on the Open Science Framework (OSF Registration DOI: 10.17605/OSF.IO/5S9PY) to ensure transparency and methodological rigor throughout the review process.

## 2 Materials and methods

This systematic review with meta-analysis was conducted following the Preferred Reporting Items for Systematic Reviews and Meta-Analyses (PRISMA) 2020 guidelines (Page et al., 2021). The review protocol was prospectively registered on the Open Science Framework (OSF) Registries (Registration DOI: 10.17605/OSF.IO/5S9PY) on April 26, 2026, prior to data extraction and analysis.

### 2.1 Eligibility criteria

The eligibility criteria were defined using the PECO (Population, Exposure, Comparison, Outcome) framework adapted for observational studies in plant genetic resources (**Table 1**).

**Table 1:** PECO framework for the systematic review.

PECO element	Definition
Population (P)	*Colocasia esculenta* (L.) Schott accessions, wild, cultivated, or landrace populations sampled across any African country, without restriction on botanical variety (*esculenta* or *antiquorum*) or ploidy level.
Exposure (E)	Characterization of genetic diversity using one or more of the following approaches: (i) agromorphological (using standardized descriptors such as IPGRI/Bioversity); (ii) biochemical (nutritional or anti-nutritional profiling using validated analytical methods); (iii) molecular (using SSR, SNP, GBS, RAPD, ISSR, AFLP, or DNA sequencing markers).
Comparison (C)	Between-study comparisons of: (i) temporal evolution of characterization approaches (2010–2026); (ii) methodological differences across marker systems and descriptors; (iii) geographic coverage of African regions and countries; (iv) diversity estimates across marker technologies (SSR versus SNP/GBS).
Outcome (O)	Quantitative diversity metrics extracted from included studies: expected heterozygosity (He), observed heterozygosity (Ho), polymorphism information content (PIC), number of alleles per locus (Na), fixation index (Fst), Shannon diversity index, and coefficient of variation (CV%). Secondary outcomes include the discriminating power of markers and descriptors, methodological quality scores, and geographic coverage of studies.

Studies were included if they met all of the following criteria: (I1) original research articles reporting quantitative characterization data of *C. esculenta*; (I2) study populations entirely or partly from Africa; (I3) use of at least one of the three characterization approaches (agromorphological, biochemical, molecular); (I4) published between January 2010 and April 2026; (I5) written in English; (I6) published in peer-reviewed journals or as theses with accessible data.

Studies were excluded if they met any of the following: (E1) editorials, conference abstracts, or opinion pieces without original data; (E2) studies conducted entirely outside Africa; (E3) studies on species other than *C. esculenta*; (E4) studies reporting only qualitative descriptions without quantitative data; (E5) duplicate publications of the same dataset; (E6) non-English language articles; (E7) studies published before 2010.

Review articles identified during screening were retained separately for the narrative synthesis but excluded from the quantitative meta-analysis.

### 2.2 Information sources and search strategy

A comprehensive search was conducted across five electronic databases: PubMed, Google Scholar, AGRIS (FAO), African Journals Online (AJOL), and OpenAlex. These databases were selected to maximize coverage of both international and African-specific literature on taro characterization. Scopus and Web of Science were not used due to lack of institutional access; OpenAlex was included as a complementary open-access alternative with broad indexing coverage.

The search was performed between March and April 2026. The generic search equation combined controlled vocabulary and free-text terms: (“*Colocasia esculenta* “ OR taro OR cocoyam OR dasheen OR eddoe) AND (characterization OR diversity OR genetic OR morphological OR agromorphological OR biochemical OR nutritional OR molecular OR SSR OR SNP OR AFLP OR RAPD OR ISSR) AND (Africa OR “West Africa” OR “East Africa” OR “Southern Africa” OR Nigeria OR Ghana OR Ethiopia OR Kenya OR Cameroon OR “South Africa” OR “Côte d’Ivoire” OR Tanzania OR Uganda OR Malawi). This equation was adapted for each database according to its specific syntax and search capabilities. The complete search strategies per database are provided in **Supplementary Material S2**.

The literature search was restricted to English-language publications, which is acknowledged as a methodological limitation. While this restriction may have led to the omission of studies published in French or Portuguese, the majority of peer-reviewed African taro research is published in international English-language journals, reflecting the norms of academic evaluation systems that reward publication in English-language literature. Nevertheless, an unknown fraction of local scientific output including theses, national bulletins, and articles in local-language journals may not have been captured, particularly from francophone Central African countries.

### 2.3 Study selection

All references retrieved from the five databases were exported to Zotero (version 6.0) for reference management and initial deduplication. The deduplicated set was then imported into Rayyan (Ouzzani et al., 2016), a web-based tool for systematic review screening, where further duplicates were identified and removed.

Screening was performed in two phases. In Phase 1 (title and abstract screening), the 198 deduplicated references were assessed by two reviewers (KRJU and NK) independently against the predefined inclusion and exclusion criteria. The Rayyan blind mode was enabled throughout to prevent bias during screening. Inter-rater agreement between the two reviewers was assessed using Cohen’s kappa (k) statistic and interpreted according to Landis and Koch (1977). Raw agreement between the two reviewers reached 92.4%, with a Cohen’s kappa of 0.84, corresponding to ‘almost perfect’ agreement. The 15 discrepancies were resolved through discussion between the two reviewers, with consultation of a senior co-author in cases where agreement could not be reached. In Phase 2 (full-text assessment), the same dual-reviewer procedure was applied to the 79 potentially eligible articles.

### 2.4 Data extraction

Data were extracted using a standardized extraction grid developed in Microsoft Excel, organized into six worksheets: (1) Identification, containing bibliographic details, study location, sample size, and characterization type; (2) Agromorphological data, including the number and type of descriptors, reference framework, Shannon diversity indices, coefficients of variation, PCA variance explained, and clustering results; (3) Biochemical data, including starch, protein, mineral, and oxalate contents with associated analytical methods; (4) Molecular data, including marker type and number, sample size, expected heterozygosity (He), observed heterozygosity (Ho), polymorphism information content (PIC), number of alleles per locus (Na), fixation index (Fst), and population structure results; (5) Quality assessment scores; and (6) PRISMA summary flow data.

For studies reporting diversity metrics as ranges, the midpoint was calculated as the mean estimate and the range divided by four was used as an approximate standard deviation. For studies reporting means with standard deviations, values were extracted directly. When standard errors were reported, they were converted to standard deviations using the formula. SD = SE × √n, where n is the number of loci.

### 2.5 Quality assessment

The methodological quality of included studies was assessed using an *ad hoc* quality scoring tool inspired by the Joanna Briggs Institute (JBI) Critical Appraisal Tools, adapted for characterization studies in plant genetic resources. The tool comprised five common criteria applicable to all study types and three type-specific criteria for each characterization approach.

The five common criteria were: (Q1) sample size, scored 0 (<15 accessions), 1 (15–29), or 2 (≥30); (Q2) protocol description (0/1); (Q3) geographic sampling, scored 0 (single site), 1 (multiple sites within one region), or 2 (multiple regions or countries); (Q4) appropriateness of statistical analyses (0/1); and (Q5) reproducibility of methods and data availability (0/1).

For agromorphological studies, three additional criteria were: (Q6a) number of descriptors ≥15 (0/1); (Q6b) use of a standardized framework such as IPGRI/Bioversity International descriptors (0/1); and (Q6c) multi-season or multi-site replications (0/1). For biochemical studies: (Q7a) number of components analyzed ≥5 (0/1); (Q7b) use of validated analytical methods such as AOAC or HPLC (0/1); and (Q7c) analytical replications (0/1). For molecular studies: (Q8a) number of loci ≥10 (0/1); (Q8b) DNA quality verification reported (0/1); and (Q8c) primer sequences or references provided (0/1).

The total quality score ranged from 0 to 10 (common criteria: 0–6; type-specific: 0–3). Studies were classified as High quality (score ≥8), Medium quality (5–7), or Low quality (<5). Quality scores were used descriptively and in sensitivity analyses but did not serve as exclusion criteria.

### 2.6 Data synthesis

#### 2.6.1 Narrative synthesis

A narrative synthesis was conducted for all 64 included studies (44 original research articles and 20 review articles). The synthesis described the temporal evolution of characterization approaches, the geographic distribution of studies across African regions, the types of molecular markers and descriptors used, and the main findings of each characterization dimension. Review articles contributed to the contextual discussion but were excluded from the quantitative meta-analysis.

#### 2.6.2 Quantitative meta-analysis

Meta-analyses were performed on molecular diversity metrics for which at least three independent studies reported comparable estimates: expected heterozygosity (He), polymorphism information content (PIC), and number of alleles per locus (Na). For each variable, a random-effects model was fitted using the restricted maximum likelihood (REML) estimator, as recommended for heterogeneous datasets with few studies (Viechtbauer, 2010). The effect measure was the raw (untransformed) mean, pooled across studies using inverse-variance weighting.

Heterogeneity was assessed using the Q statistic of Cochran and the I^2^ index (Higgins et al., 2003). The I^2^ values were interpreted as low (<25%), moderate (25–75%), or high (>75%). Prediction intervals were computed to reflect the expected range of true effects in future similar studies. The between-study variance (τ^2^) was estimated as part of the REML procedure.

Given the anticipated methodological heterogeneity across studies, marker-stratified meta-analyses were performed for each diversity metric considering SSR-based studies and SNP/GBS-based studies as independent primary analyses rather than only as subgroups within a single pooled model. This stratified approach was preferred because SSR and SNP/GBS markers differ fundamentally in their mutation rates, allelic spectra, and information content, making the interpretation of a single pooled estimate potentially uninformative. Statistical comparison between the stratified pooled estimates was performed using a Z-test for the difference between two means with independent standard errors.

Additional subgroup analyses were conducted to explore other potential sources of heterogeneity: (i) by African region (West, East, Southern Africa), and (ii) by publication period (2010–2015, 2016–2020, 2021–2026). A mixed-effects model was used for subgroup comparisons, with the subgroup variable treated as a moderator.

Univariate random-effects meta-regression was conducted for He to quantify the influence of study-level moderators on the pooled estimates. Four moderators were considered: marker type (SSR vs SNP/GBS vs Other), number of loci, number of accessions, and African region. Meta-regression models were fitted using the metafor package (Viechtbauer, 2010) with REML estimation, and the proportion of heterogeneity explained by each moderator (R^2^) was computed alongside the test statistic Q_M. Standard methodological recommendations suggesting a minimum of ten studies per moderator (Borenstein et al., 2009) were considered when interpreting meta-regression results.

Publication bias was assessed visually using funnel plots (standard error vs. effect size) and statistically using Egger’s regression test for asymmetry (Egger et al., 1997), applied when at least 10 studies were available for a given variable.

Sensitivity analyses were performed using two complementary approaches. First, leave-one-out analyses were conducted, in which each study was sequentially removed and the meta-analysis re-run to assess the influence of individual studies on the pooled estimate. Second, quality-based sensitivity analyses were performed by excluding studies classified as Low quality (score < 5) and comparing the resulting pooled estimates with those obtained from the full dataset. A quantitative meta-analysis of biochemical parameters was not performed. Although the five included biochemical studies reported overlapping parameters (starch, protein, and oxalate content), they employed heterogeneous analytical methods. They also reported results on inconsistent bases and frequently omitted measures of dispersion required for meta-analytical pooling. Pooling estimates obtained under such methodologically incompatible conditions would produce a statistically defined but biologically uninterpretable summary. We therefore retained a narrative synthesis for the biochemical dimension. The standardization of analytical protocols is highlighted in the Discussion as a priority for future work in this area.

### 2.7 Software

All statistical analyses were conducted in R version 4.3.3 (R Core Team, 2024) using the meta package (version 7.0-0; Balduzzi et al., 2019) for random-effects meta-analyses, forest plots, and funnel plots, and the metafor package (version 4.6-0; Viechtbauer, 2010) for meta-regression and advanced diagnostics. Reference management was performed with Zotero (version 6.0), and study screening with Rayyan (Ouzzani et al., 2016). Data extraction and quality scoring were performed in Microsoft Excel.

The complete R code, data extraction grids, and supplementary materials are available on the OSF project page (DOI: 10.17605/OSF.IO/5S9PY).

## 3 Results

### 3.1 Study selection and screening process

The database search yielded a total of 222 records across the five electronic sources consulted. Google Scholar contributed the largest share with 152 references, followed by OpenAlex (26), AJOL (25), PubMed (17), and AGRIS (2). After importing all records into Zotero and subsequently into Rayyan, 24 duplicates were identified and removed, leaving 198 unique references for screening.

During the first phase of screening, titles and abstracts were assessed against the predefined eligibility criteria. Of the 198 references screened, 118 were excluded because they did not meet the inclusion criteria. The most frequent reasons for exclusion at this stage were studies conducted entirely outside Africa, absence of characterization data, and studies focusing on species other than *Colocasia esculenta.* One record was initially classified as “maybe” and was subsequently resolved as included after full-text examination. This left 79 references for full-text assessment.

In the second phase, 15 articles were excluded after full-text evaluation. The reasons for exclusion included the absence of quantitative characterization data, geographic scope limited to regions outside Africa, taxonomic focus on related Araceae species rather than *C. esculenta*, and overlap with previously included datasets from the same research groups. The complete PRISMA flow diagram summarizing the selection process is presented in **Figure 1**.

**Figure 1:**
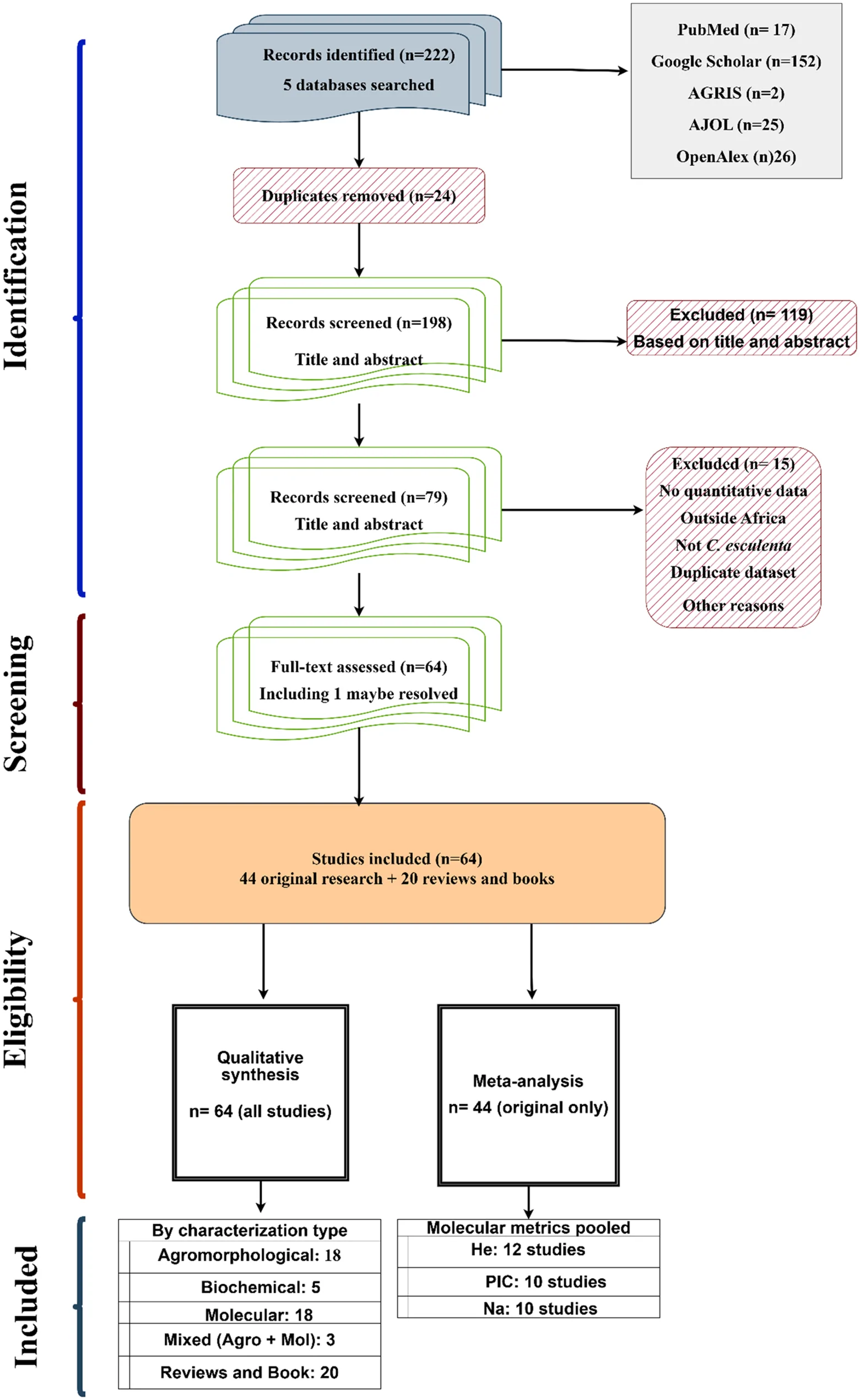
PRISMA 2020 flow diagram of the study selection process. The diagram illustrates the four phases of the systematic review: identification (records identified from five electronic databases: PubMed, Google Scholar, AGRIS, AJOL, OpenAlex; n = 222 records), screening (title and abstract screening after duplicate removal, performed by two independent reviewers in blind mode using Rayyan; Cohen’s kappa = 0.84), eligibility (full-text assessment against pre-defined inclusion criteria specified in the PECO framework), and inclusion (n = 64 studies retained, comprising 44 original research articles and 20 review articles). Exclusion reasons at each stage are documented, with the main exclusions being non-African populations, non-Colocasia species, and studies without characterization data.

Ultimately, 64 studies were retained for the review. Among these, 44 were original research articles contributing primary data, while 20 were review articles that provided contextual information and were used exclusively in the narrative synthesis. The original research articles were distributed as follows: 18 agromorphological studies, 18 molecular studies, 5 biochemical studies, and 3 studies combining agromorphological and molecular approaches. The entire screening process, conducted in four sessions using Rayyan’s blind mode, required approximately one hour and forty-four minutes.

### 3.2 Characteristics of included studies

#### 3.2.1 Temporal distribution

The 44 original research articles spanned the period from 2010 to 2025, with a noticeable increase in publication frequency over time. During the first half of the study period (2010–2015), relatively few characterization studies were published, reflecting the nascent state of taro research in Africa. The period from 2016 to 2020 saw a marked increase, particularly in molecular studies employing microsatellite markers. The most recent period (2021–2026) witnessed a diversification of approaches, with the emergence of next-generation sequencing-based methods and a growing interest in multi-trait characterization. This temporal pattern mirrors broader trends in orphan crop research across the continent, where increased funding and awareness have accelerated genetic resource documentation (Mabhaudhi et al., 2019; Tadele, 2019).

#### 3.2.2 Geographic distribution

The geographic coverage of included studies was uneven across African regions. West Africa was the first most represented region, with studies from Nigeria, Ghana, Côte d’Ivoire, and Togo. East Africa, particularly Ethiopia and Kenya, contributed large number of studies, reflecting sustained research efforts at institutions such as the Jimma Agricultural Research Center and Masinde Muliro University of Science and Technology. Southern Africa contributed through work conducted in South Africa and Malawi, primarily led by the Agricultural Research Council. Central and North Africa remained conspicuously underrepresented, with no original characterization studies identified from these subregions during the review period (**Figures 2**, **3**).

**Figure 2:**
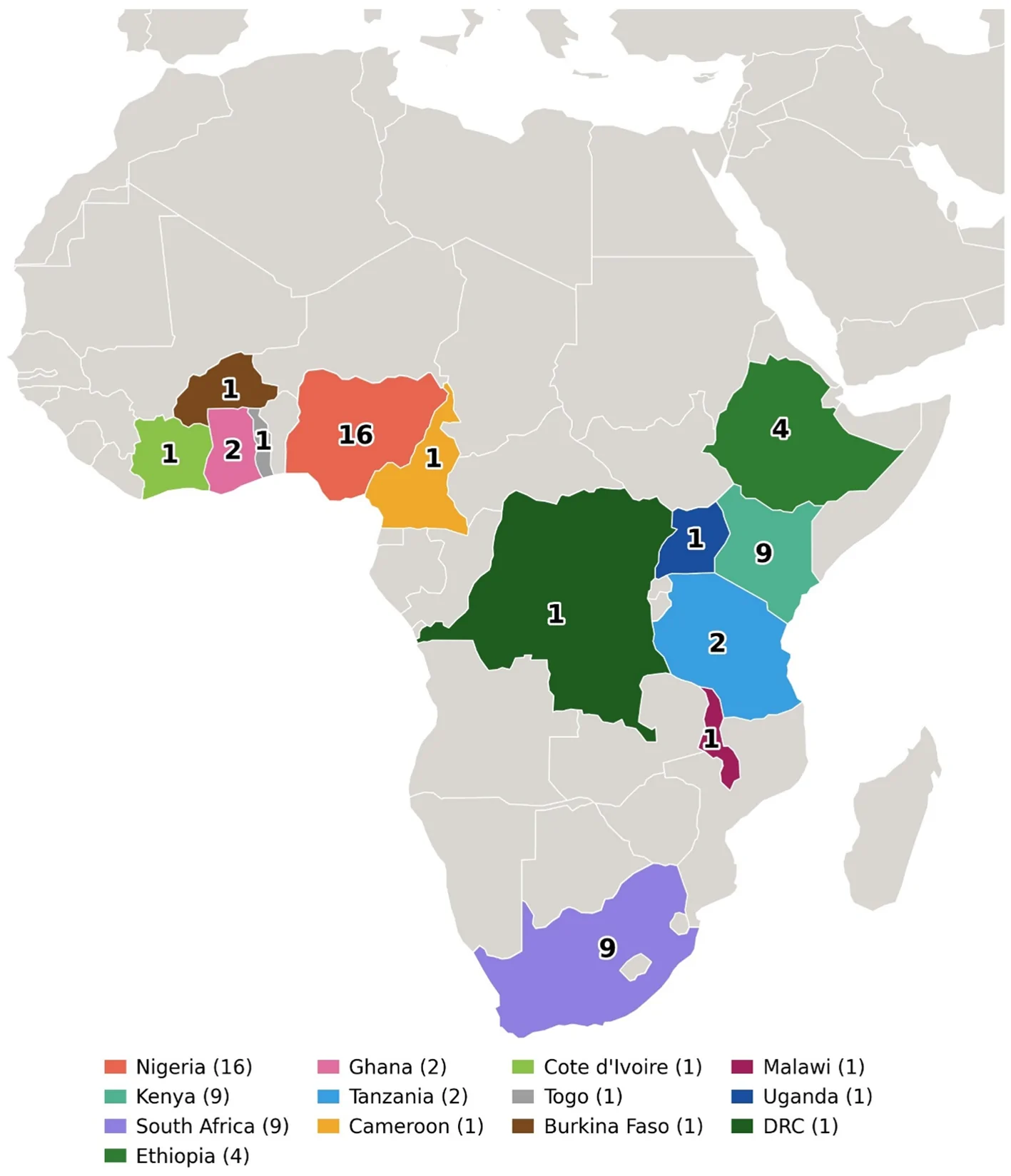
Geographic distribution of the 44 original research articles on the characterization of *Colocasia esculenta* in Africa (2010–2025), mapped by country of origin of the study populations. Colour intensity reflects the number of studies per country, illustrating the strong concentration of research in Nigeria, Kenya, South Africa, and Ethiopia, and the marked underrepresentation of Central and North Africa.

**Figure 3:**
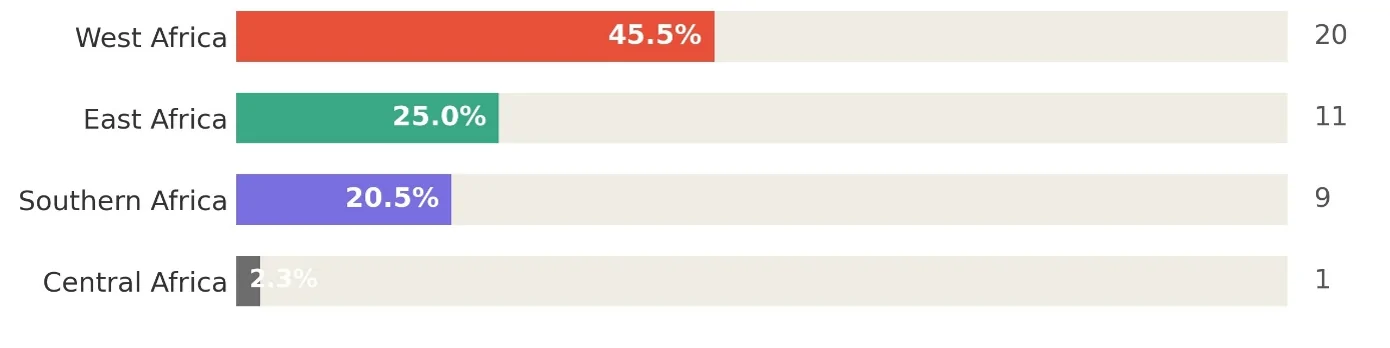
Distribution of the 44 original research articles on the characterization of *Colocasia esculenta* by African sub-region (West, East, Southern, Central, and North Africa). Each bar shows the number of studies conducted per sub-region, with West Africa (primarily Nigeria) and East Africa (Kenya, Ethiopia) accounting for the majority of the corpus. Central Africa and North Africa are markedly underrepresented, with each contributing fewer than five studies. This uneven distribution highlights a persistent geographic gap in African taro research.

This geographic imbalance has practical implications for the comprehensiveness of available genetic diversity data. Large taro-producing countries such as Cameroon, the Democratic Republic of Congo, and Madagascar where taro plays a significant dietary role were either absent or poorly represented in the literature. Similar geographic gaps have been documented in systematic reviews of other root and tuber crops in Africa (Oladimeji et al., 2022; Chivenge et al., 2015).

#### 3.2.3 Sample sizes and experimental designs

The number of accessions evaluated per study varied considerably, ranging from fewer than 10 in some preliminary assessments to over 400 in large-scale diversity panels. Across the original studies included in this review, sample sizes (number of accessions characterized per study) varied substantially. The overall median sample size across the studies with identifiable characterization data was 26 accessions (interquartile range: 25 to 95; minimum: 20, maximum: 321), reflecting the wide range of study scales encountered in the literature, from small case studies of local landraces to large-scale continental collections. Sample sizes varied by characterization approach: agromorphological studies (k = 14) had a median of 31 accessions (IQR: 25-97; range: 20-321); molecular studies (k = 15) had a median of 26 accessions (IQR: 25-95; range: 20-321); and biochemical studies (k = 4) had a median of 190 accessions (IQR: 77-292; range: 14-321). This heterogeneity in study scale has important implications for the representativeness of individual studies and for the interpretation of pooled estimates, as smaller studies tend to over-represent local diversity while larger studies provide broader geographic coverage but less within-site resolution. Agromorphological studies tended to employ randomized complete block designs with two to three replications at a single site, although a few notable exceptions involved multi-location trials across diverse agro-ecological zones (Gerrano et al., 2019; Debela et al., 2025). Molecular studies typically analyzed between 20 and 100 accessions using panels of 10 to 30 markers. The biochemical studies generally involved smaller sample sizes, reflecting the resource-intensive nature of laboratory analyses.

### 3.3 Quality assessment

The methodological quality of the 44 original research articles was evaluated using the *ad hoc* scoring tool described in the methods section. The distribution of quality scores by characterization type is presented in **Table 2**. Of these, 32 studies (72.7%) were classified as High quality (score ≥ 8), 8 studies (18.2%) as medium quality (score 5–7), and 4 studies (9.1%) as low quality (score < 5). The most common methodological limitations were the absence of multi-season or multi-site replications in agromorphological studies, small sample sizes in biochemical analyses, and insufficient reporting of DNA quality verification procedures in some molecular studies.

**Table 2:** Methodological quality assessment of the 44 original research articles included in the systematic review, classified by characterization type.

Characterization type	n	High (≥8)	Medium (5–7)	Low (<5)	Mean score
Agromorphological	18	10 (55.6%)	5 (27.8%)	3 (16.7%)	7.0
Biochemical	5	3 (60.0%)	1 (20.0%)	1 (20.0%)	7.4
Molecular	18	16 (88.9%)	2 (11.1%)	0 (0.0%)	8.6
Mixed (Agromorphological + Molecular)	3	3 (100.0%)	0 (0.0%)	0 (0.0%)	11.7
TOTAL	44	32 (72.7%)	8 (18.2%)	4 (9.1%)	8.0

Quality scores were computed using an ad hoc scoring tool adapted from the Joanna Briggs Institute (JBI) Critical Appraisal Tools, comprising five criteria common to all study types (sample size, protocol description, geographic sampling, statistical analyses, reproducibility) and three type-specific criteria for each characterization approach. The total score ranges from 0 to 10. Studies were classified as High quality (score ≥ 8), Medium quality (5–7), or Low quality (< 5). Percentages refer to the proportion of studies within each characterization type.

High-quality studies were characterized by large and geographically representative sample collections, rigorous experimental designs, comprehensive statistical analyses, and transparent reporting of methods and data. Studies classified as Low quality typically involved small convenience samples, limited geographic coverage, and incomplete descriptions of analytical procedures. No studies were excluded on the basis of quality scores, but these ratings were incorporated into the sensitivity analyses of the meta-analysis.

### 3.4 Agromorphological characterization

Eighteen original studies reported agromorphological characterization data for *C. esculenta* populations in Africa. The number of descriptors employed ranged from 10 to 30 per study, with the majority drawing on the standardized descriptor lists published by the International Plant Genetic Resources Institute, now Bioversity International (IPGRI, 1999). Across the reviewed studies, a total of approximately 45 distinct morphological and agronomic traits were documented, encompassing foliar characters (leaf shape, size, color, venation pattern, petiole attributes), subterranean traits (corm shape, size, weight, flesh color, cortex color, branching pattern), and yield-related parameters (number of cormels, corm weight, total yield per plant).

Among the most frequently assessed traits, corm shape consistently emerged as one of the most polymorphic and discriminating characters, with Shannon diversity indices reaching 1.40 in Kenyan germplasm collections (Akwee and Palapala, 2017). Corm cortex color and corm flesh color also showed substantial variation, with diversity indices of 1.20 and 0.97, respectively. Petiole junction color was another highly variable trait, particularly useful for distinguishing accessions at the vegetative stage prior to harvest.

In terms of yield performance, the studies revealed considerable variation among African taro accessions. In Kenya, estimated yields of approximately 5.8 tonnes per hectare were reported for local landraces (Akwee and Palapala, 2023), while in South Africa, corm weights ranged from 227 to 780 grams per plant across 29 Amadumbe genotypes evaluated at two locations (Gerrano et al., 2019). Ethiopian studies documented even greater variation, with 100 accessions from southwestern Ethiopia displaying yield ranges spanning several orders of magnitude and broad-sense heritability estimates of up to 30% for petiole length (Tewodros, 2013).

Multivariate analyses were commonly employed to explore patterns of morphological diversity. Principal component analysis across the reviewed studies typically showed that the first two to three components explained between 50% and 75% of the total variation. Plant height, leaf dimensions, and corm weight were recurrently identified as the traits contributing most heavily to the first principal component, suggesting that vegetative vigor and underground storage capacity are the primary axes of phenotypic differentiation in African taro germplasm.

Cluster analyses, performed using UPGMA or Ward’s method in most studies, generally identified three to seven distinct morphological groups. The clustering patterns were often associated with geographic origin, botanical variety (dasheen versus eddoe types), and end-use characteristics. In Ghana, Boampong et al. (2018) identified five distinct clusters among 18 accessions of diverse geographic origin, with accessions from Samoa and Malaysia grouping separately from local Ghanaian landraces. A particularly informative study by Debela et al. (2025) employed AMMI and GGE biplot analyses across six environments in southwestern Ethiopia, identifying genotype JD/Ce23 as the most stable and highest-yielding among 10 tested genotypes, with a mean yield of 37.56 tonnes per hectare.

### 3.5 Biochemical characterization

Five original studies provided data on the biochemical composition of African taro accessions. The nutritional profiles reported across these studies revealed substantial variation in key constituents, particularly starch content, crude protein, mineral composition, and anti-nutritional factors such as oxalates and phytates.

Starch content, the predominant component of taro corms, ranged from approximately 15% to 28% on a dry weight basis across the reviewed studies. Crude protein content was generally low, ranging from 1.5% to 7%, consistent with the profile expected for starchy root and tuber crops. Mineral analyses revealed that taro corms are notable sources of potassium, calcium, magnesium, and iron, with considerable inter-accession variability that could be exploited in biofortification programs.

Oxalate content, a major concern for taro utilization due to its effects on palatability and calcium bioavailability, showed wide variation among accessions. Some studies reported that traditional processing methods such as boiling and fermentation can reduce oxalate levels by 40% to 70%, suggesting that targeted selection for low-oxalate genotypes combined with appropriate post-harvest handling could significantly improve the nutritional quality of taro-based foods (Adane et al., 2013).

The limited number of biochemical studies identified in this review highlights a significant gap in the literature. While taro is increasingly recognized for its nutritional value and potential contributions to food security, systematic biochemical profiling across diverse African germplasm collections remains scarce. This contrasts with the relatively more abundant molecular and agromorphological data, suggesting that biochemical characterization has not received commensurate research attention.

### 3.6 Molecular characterization

#### 3.6.1 Evolution of molecular markers

The 18 molecular characterization studies and 3 mixed agromorphological-molecular studies collectively documented the genetic diversity of African taro using a range of marker systems (**Figure 4**). The most widely employed markers were simple sequence repeats (SSR or microsatellites), used in the majority of studies. This predominance reflects the codominant nature, high reproducibility, and locus-specific information provided by SSR markers, which make them particularly suitable for diversity assessment in vegetatively propagated crops (Mace et al., 2006).

**Figure 4:**
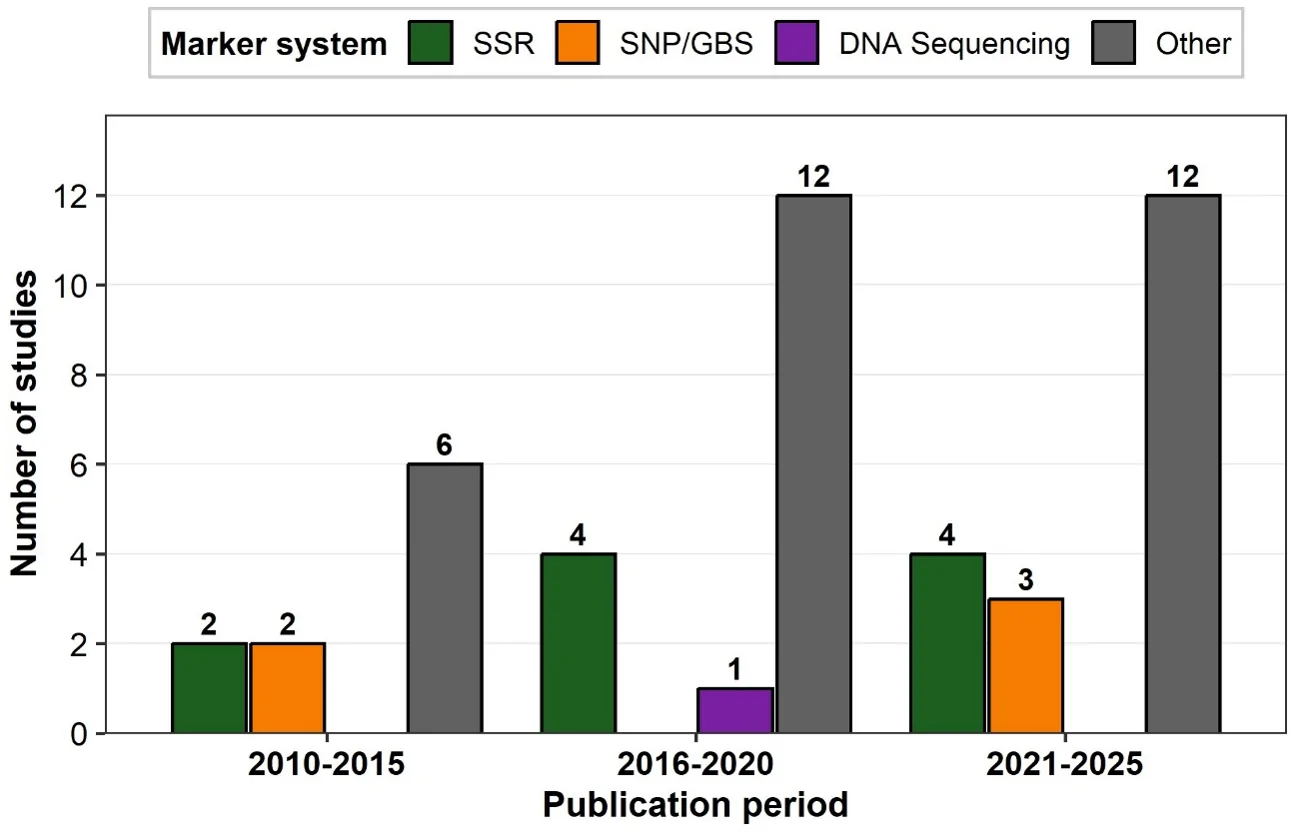
Temporal evolution of molecular marker systems used for the characterization of *Colocasia esculenta* in Africa between 2010 and 2025, based on the 17 original studies reporting molecular characterization data. The stacked bars show the proportion of studies employing each marker type (SSR, SNP/GBS, RAPD, ISSR, Sequencing, and Other/mixed) across three publication periods (2010–2015, 2016–2020, 2021–2025). The plot highlights the methodological shift from dominant markers in the early 2010s toward SSR-based characterization (58.8% of recent studies) and the more recent emergence of high-throughput SNP/GBS approaches (29.4%), reflecting the wider trend of technology adoption in African plant genetic resources research.

A clear temporal shift in marker technology was observable across the review period. Studies published between 2010 and 2015 frequently relied on dominant markers such as RAPD and AFLP, which provide genome-wide coverage but limited allelic resolution. From 2016 onward, SSR markers became the standard tool, with several studies using panels developed specifically for *Colocasia* (Mace and Godwin, 2002; Singh et al., 2012). More recently, single nucleotide polymorphism (SNP) markers generated through genotyping-by-sequencing (GBS) platforms have begun to appear in the literature, offering dramatically higher marker densities and enabling more detailed population genomic analyses (Oladimeji et al., 2022).

#### 3.6.2 Diversity metrics across studies

Expected heterozygosity (He), the most commonly reported measure of within-population genetic diversity, was available from 12 independent study-marker combinations. Values ranged from as low as 0.12 in studies using RAPD markers on small collections to 0.74 in SSR-based analyses of large and geographically diverse panels. The substantial range of He values across studies reflects differences in marker informativeness, sample composition, and geographic scope.

Polymorphism information content (PIC), reported in 10 studies, showed a similarly broad range. SSR-based studies generally reported PIC values between 0.30 and 0.65, while studies using dominant markers tended to report lower values. The number of alleles per locus (Na) ranged from 2 to approximately 8 across studies, with higher values typically associated with SSR markers applied to larger and more geographically diverse collections.

Population differentiation, assessed through Fst or analogous statistics, was reported in fewer studies. Where available, Fst values ranged from moderate (0.05–0.15) to high (> 0.15), suggesting substantial genetic structuring among taro populations across different regions of Africa. Analysis of molecular variance (AMOVA), when performed, consistently attributed the largest proportion of genetic variation to differences within populations rather than among populations, a pattern commonly observed in vegetatively propagated crop species (Macharia et al., 2014; Grimaldi et al., 2018).

### 3.7 Meta-analysis results

#### 3.7.1 Expected heterozygosity

The random-effects meta-analysis of expected heterozygosity, based on 12 independent estimates from molecular studies, yielded a pooled value of He = 0.40 (95% CI: 0.32–0.48). The between-study heterogeneity was very high, as indicated by an I^2^ value of 99.5% and a highly significant Q statistic (Q = 2348.87, p < 0.0001). The between-study variance was estimated at τ^2^ = 0.019 (**Figure 5**).

**Figure 5:**
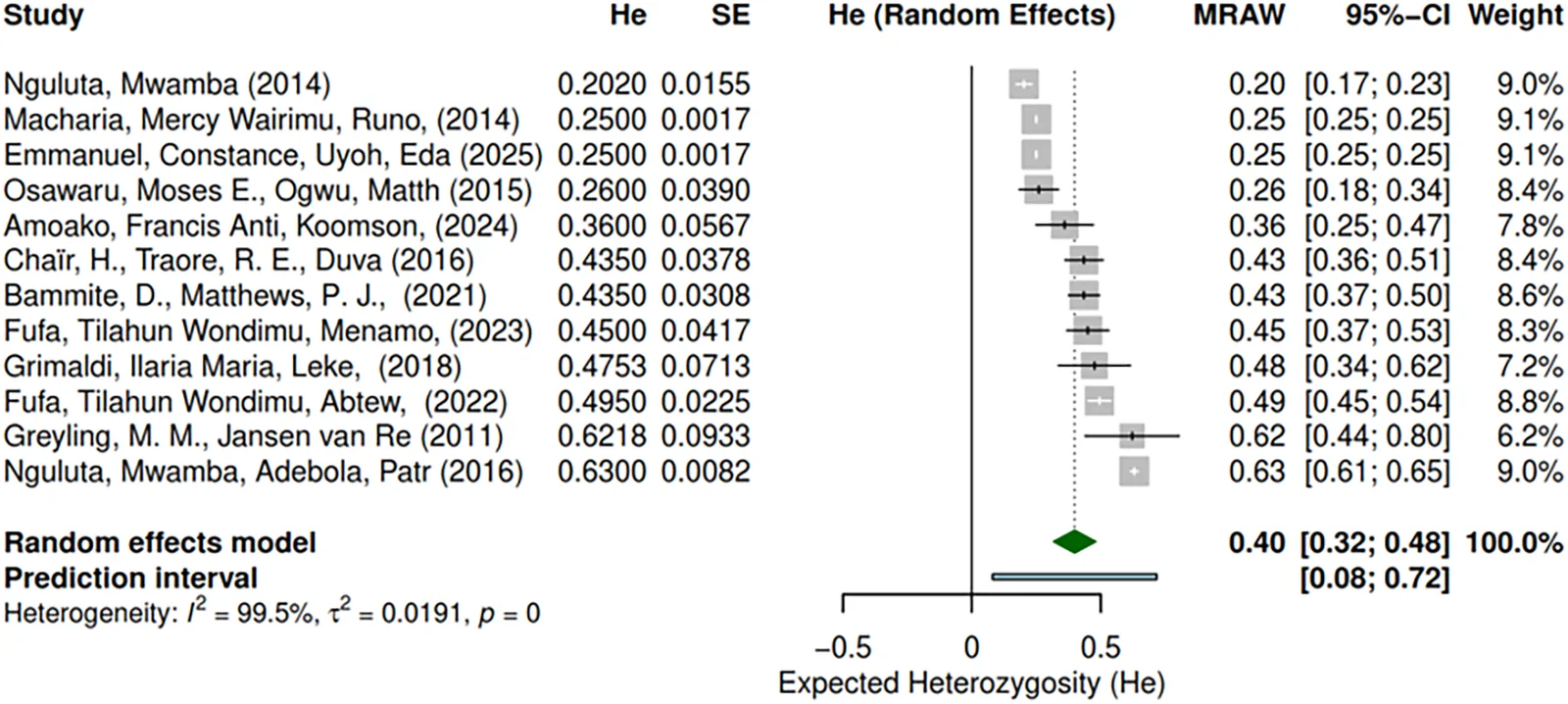
Forest plot of the random-effects meta-analysis of expected heterozygosity (He) in African *Colocasia esculenta*, based on 12 independent estimates. Individual study estimates are shown as squares with size proportional to study weight and horizontal lines representing 95% confidence intervals; the pooled random-effects estimate (He = 0.40; 95% CI: 0.32–0.48; 95% prediction interval: 0.08–0.72) is represented by the diamond at the bottom. Between-study heterogeneity was very high (I^2^ = 99.5%; τ^2^ = 0.019).

The pooled estimate of He = 0.40 indicates a moderate level of genetic diversity in African taro populations. For context, this value falls within the range typically reported for vegetatively propagated tropical crops. Comparable meta-analytical estimates for other clonally propagated species have yielded similar values: cassava populations in Africa generally show He values between 0.35 and 0.55 (Kawuki et al., 2013), while yam (*Dioscorea* spp.) diversity studies report He values ranging from 0.30 to 0.60 depending on the species and geographic scope (Scarcelli et al., 2019). The moderate He observed here suggests that despite its predominantly clonal propagation, African taro has maintained a meaningful reservoir of allelic diversity, possibly sustained by occasional sexual reproduction, farmer-mediated selection, and geographic isolation.

The very high I^2^ value of 99.5% warrants careful interpretation. While this figure indicates that virtually all observed variation among studies reflects genuine differences rather than sampling error, such extreme heterogeneity is not unexpected given the diversity of marker systems, sample sizes, and geographic scopes represented in the pooled dataset. Studies employing highly polymorphic SSR markers on large collections from multiple countries naturally yield higher He estimates than those using dominant markers on small local samples. To address this heterogeneity, we conducted marker-stratified analyses and meta-regression, described in the following subsections.

#### 3.7.2 Marker-stratified meta-analyses

Given the fundamental differences between SSR and SNP/GBS marker systems in terms of mutation rates, allelic spectra, and information content, we conducted separate meta-analyses for each marker type as primary analyses rather than only as subgroups within a single pooled model. This stratified approach was preferred because SSR and SNP/GBS markers differ fundamentally in their molecular basis, making the interpretation of a single pooled estimate potentially uninformative. **Table 3** summarizes the results of these marker-stratified analyses for the three-diversity metrics.

**Table 3:** Marker-stratified meta-analyses of molecular diversity metrics for *Colocasia esculenta* in Africa.

Metric	k	Pooled estimate	95% CI	95% PI	I^2^ (%)	τ^2^
He — All studies	12	0.3987	[0.317, 0.480]	[0.081, 0.717]	99.5	0.019
He — SSR only	7	0.4452	[0.331, 0.559]	[0.061, 0.829]	99.0	0.021
He — SNP/GBS only	5	0.3390	[0.233, 0.445]	[-0.023, 0.701]	97.2	0.014
PIC — All studies	10	0.4645	[0.384, 0.545]	[0.176, 0.753]	92.5	0.015
PIC — SSR only	5	0.4633	[0.376, 0.551]	[0.203, 0.724]	74.5	0.007
PIC — SNP/GBS only	5	0.4679	[0.320, 0.616]	[-0.030, 0.965]	96.0	0.026
Na — All studies	10	3.7176	[3.131, 4.304]	[1.626, 5.810]	89.6	0.766
Na — SSR only	9	3.6167	[2.989, 4.244]	[1.452, 5.781]	85.3	0.778
Na — SNP/GBS only	<3	N/A	—	—	—	—

k, number of studies; CI, confidence interval; PI, prediction interval; I^2^, proportion of total variation due to between-study heterogeneity; τ^2^, between-study variance. All estimates obtained under a random-effects model with restricted maximum likelihood (REML) estimation. Highlighted rows (“All studies”) represent the overall pooled analyses; unhighlighted rows represent the marker-stratified analyses. Na SNP/GBS only was not calculated due to insufficient studies (k < 3), reflecting the fact that number-of-alleles-per-locus metrics are rarely reported for biallelic SNP markers.

For He, the SSR-only meta-analysis based on seven studies yielded a pooled estimate of 0.45 (95% CI: 0.33–0.56; 95% PI: 0.06–0.83; I^2^ = 99.0%), whereas the SNP/GBS-only meta-analysis based on five studies yielded a lower pooled estimate of 0.34 (95% CI: 0.23–0.45; 95% PI: −0.02–0.70; I^2^ = 97.2%). Although the confidence intervals of the two estimates overlap, the direction of the difference (SSR > SNP/GBS by 0.11 units) is consistent with the higher allelic resolution of codominant microsatellite markers relative to bi-allelic SNPs, and matches expectations for a vegetatively propagated crop. The formal test for the difference between the two pooled estimates did not reach statistical significance (Z = 1.33, p = 0.18), likely reflecting the limited number of studies in each subgroup (**Figures 6**, **7**).

**Figure 6:**
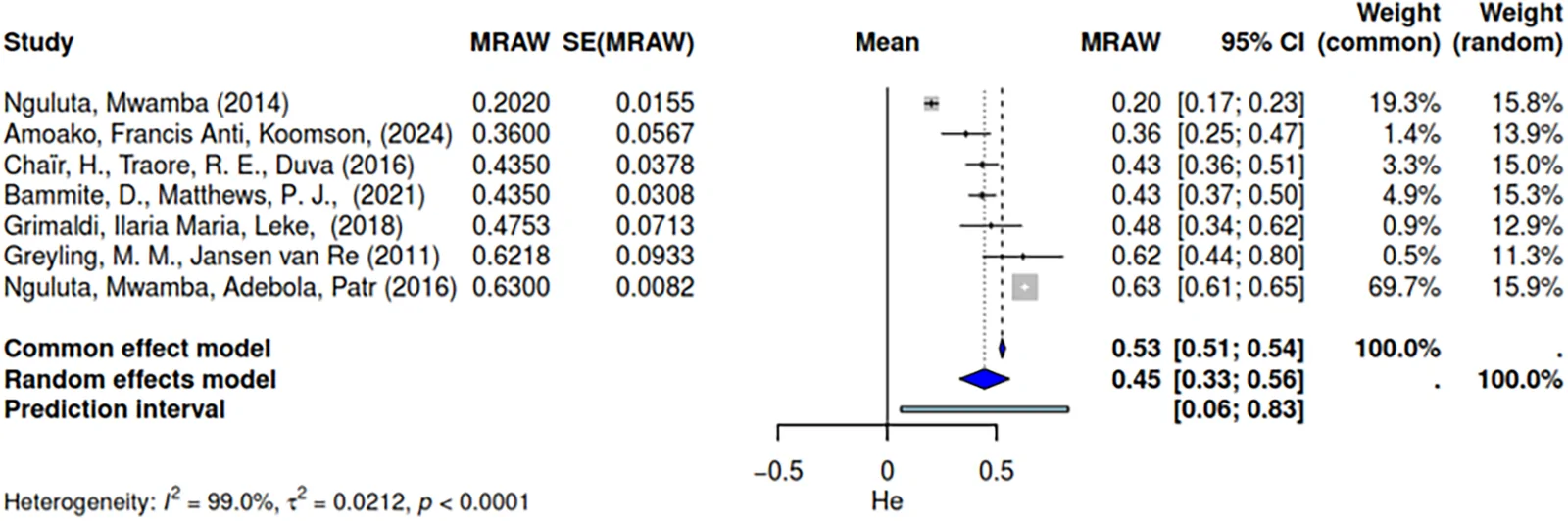
Forest plot of the SSR-only random-effects meta-analysis of expected heterozygosity (He) in African *Colocasia esculenta*, based on 7 independent studies employing simple sequence repeat (SSR) markers. Individual study estimates are shown as squares with 95% confidence intervals; the pooled random-effects estimate (He = 0.45; 95% CI: 0.33-0.56; 95% prediction interval: 0.06-0.83) is represented by the diamond at the bottom. Between-study heterogeneity remained very high (I^2^ = 99.0%). The higher pooled He estimate compared to SNP/GBS (**Figure 7**) is consistent with the higher polymorphism detection potential of codominant SSR markers.

**Figure 7:**
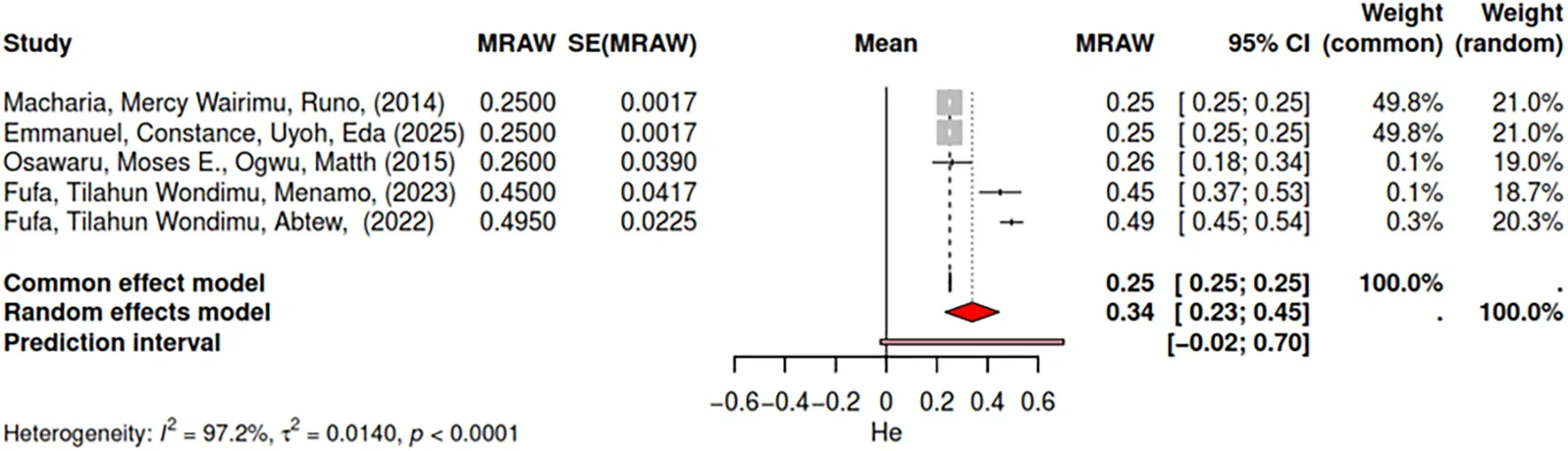
Forest plot of the SNP/GBS-only random-effects meta-analysis of expected heterozygosity (He) in African *Colocasia esculenta*, based on 5 independent studies employing single-nucleotide polymorphism (SNP) or genotyping-by-sequencing (GBS) approaches. Individual study estimates are shown as squares with 95% confidence intervals; the pooled random-effects estimate (He = 0.34; 95% CI: 0.23-0.45; 95% prediction interval: -0.02-0.70) is represented by the diamond at the bottom. Between-study heterogeneity was very high (I^2^ = 97.2%). The 0.11-unit difference between SSR and SNP/GBS pooled estimates was not statistically significant (Z = 1.33; p = 0.18).

For PIC, the two stratified analyses produced strikingly similar pooled estimates: 0.46 for SSR only (95% CI: 0.38–0.55; 95% PI: 0.20–0.72; I^2^ = 74.5%) and 0.47 for SNP/GBS only (95% CI: 0.32–0.62; 95% PI: −0.03–0.97; I^2^ = 96.0%). The near-equivalence of the pooled PIC estimates across the two marker technologies is noteworthy: it suggests that although SSR markers typically generate more alleles per locus, the practical informativeness for population differentiation (as quantified by PIC) is comparable between the two marker systems when averaged across studies. Importantly, the SSR-only analysis showed markedly lower between-study heterogeneity (I^2^ = 74.5%) than either the SNP/GBS-only analysis (I^2^ = 96.0%) or the pooled analysis (I^2^ = 92.5%), suggesting that SSR-based PIC estimates are more consistent across studies (**Figures 8**, **9**).

**Figure 8:**
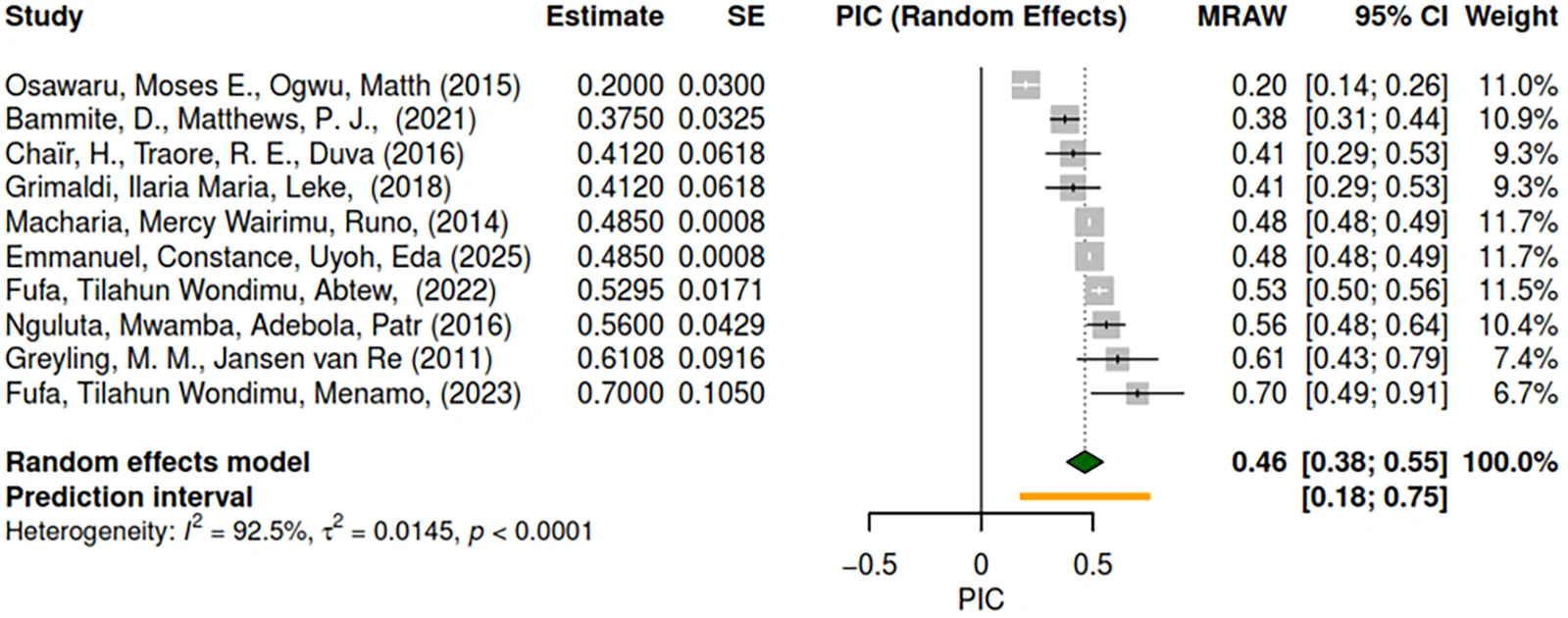
Forest plot of the random-effects meta-analysis of polymorphism information content (PIC) in African *Colocasia esculenta*, based on 10 independent estimates. Individual study estimates are shown as squares with 95% confidence intervals; the pooled random-effects estimate (PIC = 0.46; 95% CI: 0.38-0.55; 95% prediction interval: 0.18-0.75) is represented by the diamond at the bottom. Between-study heterogeneity was very high (I^2^ = 92.5%). The pooled PIC value sits near the boundary between moderately (PIC = 0.25-0.50) and highly informative (PIC > 0.50) markers as defined by Botstein et al. (1980).

**Figure 9:**
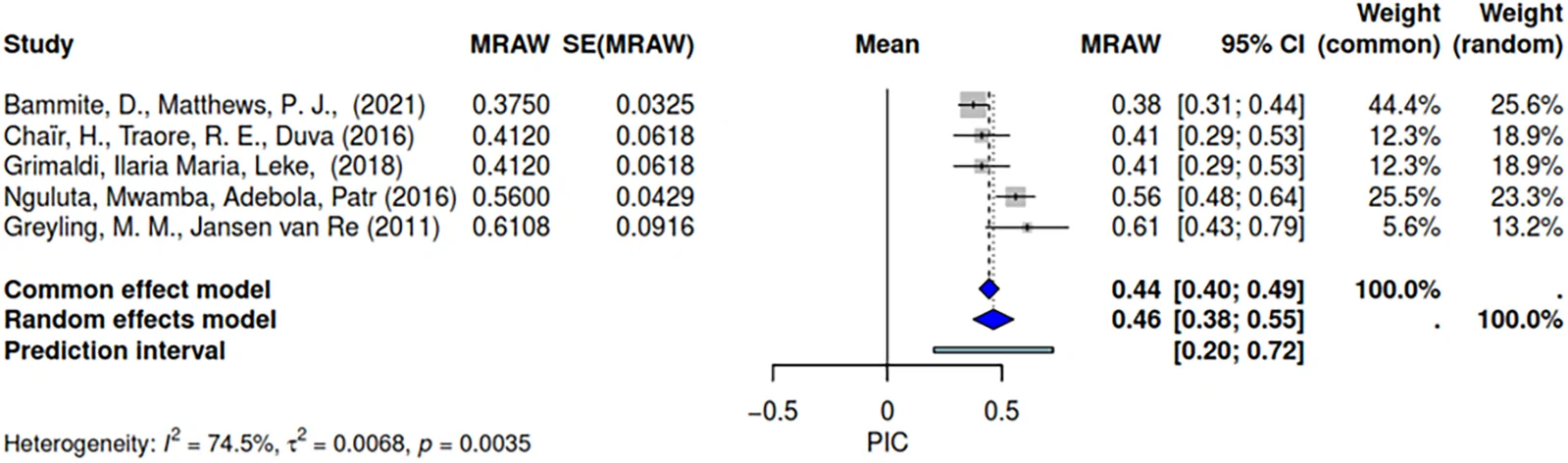
Forest plot of the SSR-only random-effects meta-analysis of polymorphism information content (PIC) in African *Colocasia esculenta*. Individual study estimates from SSR-based studies are shown as squares with 95% confidence intervals; the pooled random-effects estimate (PIC = 0.46) is represented by the diamond at the bottom. The near-identical pooled PIC values obtained for SSR (**Figure 9**) and SNP/GBS (**Figure 10**) markers indicate that PIC is largely marker-independent, unlike expected heterozygosity.

For Na, only the SSR-only meta-analysis could be performed, as fewer than three SNP/GBS studies reported this metric. The SSR-only pooled Na was 3.62 (95% CI: 2.99–4.24; 95% PI: 1.45–5.78; I^2^ = 85.3%), very close to the overall pooled estimate of 3.72. The near-absence of Na reporting in SNP/GBS studies reflects the fundamental biological difference between the two marker technologies: SNPs are predominantly biallelic and rarely characterized in terms of “number of alleles per locus,” which is a metric more directly relevant to multi-allelic microsatellite markers (**Figure 10**).

**Figure 10:**
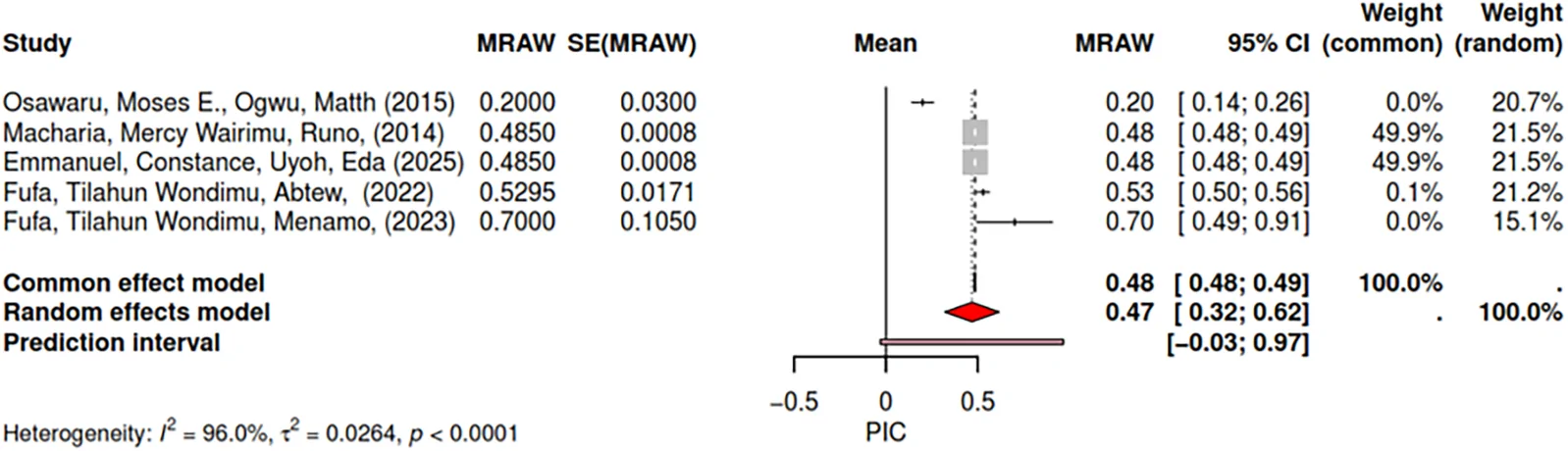
Forest plot of the SNP/GBS-only random-effects meta-analysis of polymorphism information content (PIC) in African *Colocasia esculenta*. Individual study estimates from SNP/GBS-based studies are shown as squares with 95% confidence intervals; the pooled random-effects estimate (PIC = 0.47) is represented by the diamond at the bottom. The near-identical pooled PIC estimates between SSR and SNP/GBS analyses (0.46 vs 0.47) confirm that polymorphism information content is largely marker-independent, in contrast to expected heterozygosity which showed marker-dependent variation.

Together, the stratified analyses substantially reduce methodological heterogeneity for two of the three metrics: the SSR-only I^2^ for PIC dropped from 92.5% to 74.5%, confirming that marker system is an important source of between-study heterogeneity for this metric. Forest plots for each marker-specific analysis are provided in the main figures.

#### 3.7.3 Meta-regression

To quantify the contribution of study-level moderators to the observed heterogeneity in He estimates, we performed univariate random-effects meta-regression analyses considering four moderators: marker type, number of loci, number of accessions, and African region. The results are presented in **Table 4**.

**Table 4:** Univariate random-effects meta-regression of moderators on the pooled He estimate (k = 12 studies).

Moderator	k	Q_M statistic	df	p-value	R^2^ (%)
Marker type (SSR/SNP-GBS/Other)	12	0.223	2	0.894	0
Number of loci	12	0.493	1	0.483	0
Number of accessions	12	0.566	1	0.452	0
African region	12	2.092	3	0.554	0

Q_M is the test statistic for the null hypothesis that the moderator has no effect on the pooled He estimate. R^2^ represents the proportion of the between-study heterogeneity (τ^2^) explained by the moderator. All models fitted using restricted maximum likelihood (REML) estimation. None of the four moderators reached statistical significance at the conventional p < 0.05 threshold, and none explained a measurable proportion of the between-study heterogeneity. This finding is interpreted in as reflecting the limited number of studies (k = 12) and the influence of unmeasured methodological details.

None of the four moderators tested reached statistical significance (all p > 0.05), and the R^2^ value was 0% for each moderator, indicating that none of the study-level characteristics assessed explained a measurable proportion of the between-study heterogeneity in He estimates. This result should be interpreted in the light of two important considerations.

First, the limited number of studies included in the He meta-analysis (k = 12) severely constrains the statistical power of meta-regression. Standard methodological recommendations suggest that meta-regression requires a minimum of ten studies per moderator tested (Borenstein et al., 2009), a threshold that our dataset only marginally meets for univariate models and does not meet at all for multivariate models. The non-significance of marker type in the meta-regression is therefore consistent with, rather than contradictory to, the marker-stratified analyses, which showed a difference of 0.11 units between SSR (0.45) and SNP/GBS (0.34) pooled He estimates but likewise did not reach statistical significance (Z = 1.33, p = 0.18).

Beyond the power considerations discussed above, the finding that R^2^ = 0% for all four moderators warrants a substantive interpretation. Statistically, an R^2^ estimate of 0% indicates that the tested moderators do not explain any measurable proportion of the between-study heterogeneity, which can arise from three non-mutually exclusive scenarios: (i) the moderators genuinely have no systematic influence on He estimates in the underlying population of studies; (ii) the moderators do exert an influence but the current sample size is too small to detect it (Type II error); or (iii) the true effects of the moderators are masked by residual variability from unmeasured sources that dominate the total heterogeneity. Given the extremely high residual heterogeneity (I^2^ > 99% in all four models) and the biological plausibility of at least the marker-type effect (as demonstrated by the marker-stratified analyses in Section 3.7.2), scenario (iii) appears most likely: the tested moderators (marker type, number of loci, number of accessions, and African region) are not the primary drivers of between-study heterogeneity in African taro He estimates. Instead, the variability appears to be governed largely by unmeasured methodological and biological factors, including those detailed in the paragraph below. A formal *post-hoc* power analysis conducted using the R package metapower (Griffin, 2021) confirmed that our meta-regression models had only ~15–25% power to detect a moderate moderator effect (accounting for 15% of variance) at k = 12, well below the conventional 80% threshold. This underpowered configuration limits our ability to rule out true moderator effects and reinforces the interpretation that both statistical power and unmeasured factors contribute to the observed R^2^ = 0%.

A sensitivity meta-regression restricted to studies of High and Medium methodological quality was considered but could not be meaningfully computed: excluding the four studies classified as Low quality would have reduced the dataset to k = 8, well below the minimum of ten studies per moderator recommended for reliable meta-regression modelling (Borenstein et al., 2009). This further reinforces the conclusion that the current evidence base is too limited to disentangle the sources of between-study heterogeneity through meta-regression, and that the priority for future research lies in expanding the primary literature on the molecular characterization of African taro.

Second, the residual heterogeneity remained very high in all meta-regression models (I^2^ > 99%), indicating that unobserved factors not captured by our moderators dominate the observed differences between studies. Importantly, the inability of the four measured study-level characteristics to explain the observed heterogeneity strongly suggests that unmeasured or inconsistently reported methodological details are the primary drivers of between-study variability. These unmeasured factors likely include: (i) the specific SSR primer sets used, which vary considerably across studies and differ in their allelic resolution; (ii) DNA extraction and PCR amplification protocols, which affect the reliability and reproducibility of genotyping calls; (iii) geographic sampling strategy within countries, particularly whether sampling was systematic, opportunistic, or focused on specific agroecological zones; (iv) whether accessions originated from *ex situ* genebank collections or from farmer field surveys, as these two sources typically capture different fractions of the total genetic diversity; and (v) allele-calling algorithms and quality-filtering parameters, which vary substantially between SSR and SNP/GBS studies and are rarely reported in sufficient detail to enable comparison. Together, these observations reinforce the importance of standardized reporting in future primary studies as a prerequisite for more powerful and interpretable meta-analyses in this field.

#### 3.7.4 Subgroup analyses by region and period

When stratified by African region, East African studies tended to report somewhat higher He values than those from West and Southern Africa, although the confidence intervals overlapped substantially, precluding definitive conclusions about regional differences in genetic diversity. The temporal subgroup analysis showed a slight upward trend in reported He values from earlier to more recent publication periods, likely reflecting the progressive adoption of more informative marker systems rather than actual changes in population-level diversity (**Figure 11**).

**Figure 11:**
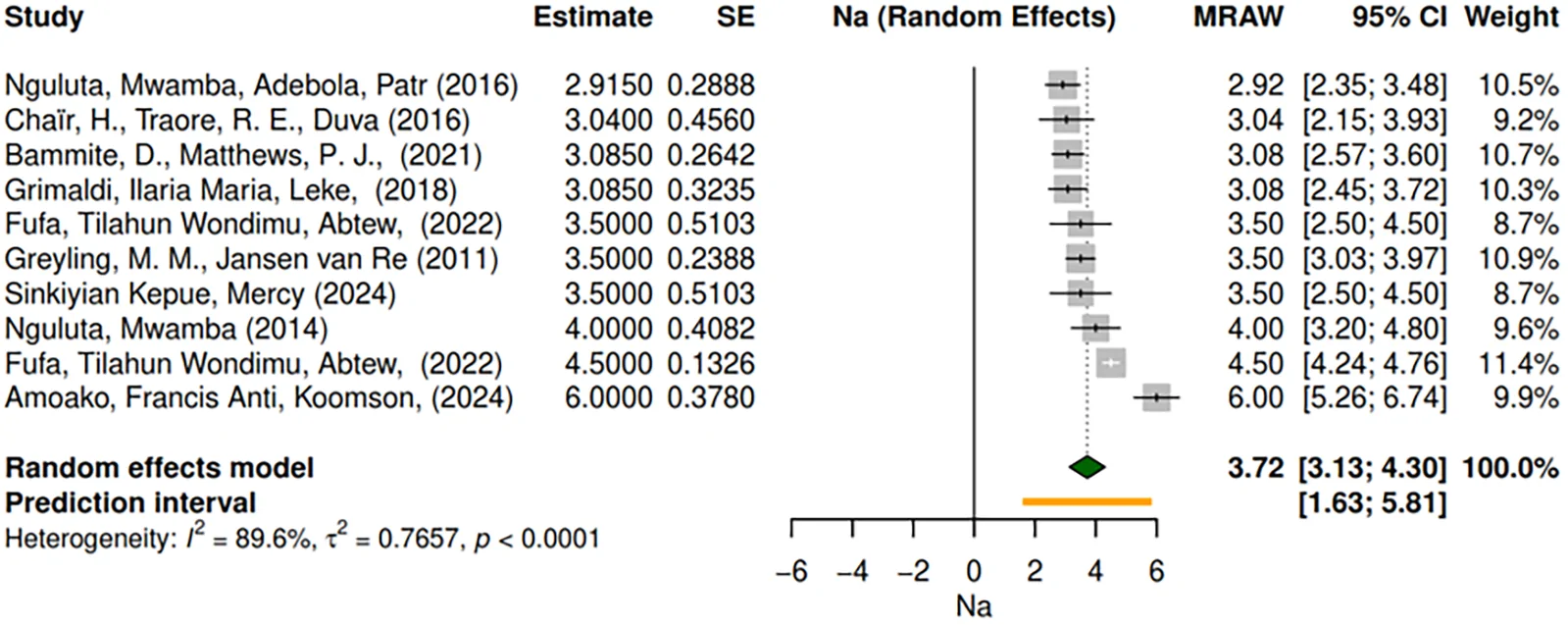
Forest plot of the random-effects meta-analysis of the mean number of alleles per locus (Na) in African *Colocasia esculenta*, based on 10 independent estimates from SSR-based studies. Individual study estimates are shown as squares with 95% confidence intervals; the pooled random-effects estimate (Na = 3.72; 95% CI: 3.13–4.30; 95% prediction interval: 1.63–5.81) is represented by the diamond at the bottom. Between-study heterogeneity was high (I^2^ = 89.6%). This average allelic richness is consistent with the intermediate reproductive strategy of taro (Hamrick and Godt, 1996).

#### 3.7.5 Polymorphism information content pooled analysis

The pooled meta-analysis of PIC values, based on 10 independent estimates, yielded a pooled PIC of 0.46 (95% CI: 0.38–0.55; 95% PI: 0.18–0.75) with high heterogeneity (I^2^ = 92.5%; Q = 120.39, p < 0.0001; τ^2^ = 0.015). According to the classification proposed by Botstein et al. (1980), PIC values above 0.50 indicate highly informative markers, values between 0.25 and 0.50 denote moderately informative markers, and values below 0.25 reflect low informativeness. The pooled estimate of 0.46 thus places the markers used across African taro studies in the upper range of the moderately informative category, approaching the threshold for high informativeness. As noted, the SSR-only and SNP/GBS-only pooled PIC values are strikingly similar (0.46 and 0.47 respectively), suggesting that PIC is a more marker-independent metric than He.

This finding suggests that the molecular markers deployed in African taro research have generally been adequate for detecting meaningful genetic variation, although there remains scope for improvement. The adoption of higher-density marker platforms such as SNP arrays or whole-genome sequencing would be expected to enhance resolution, particularly for fine-scale population structure and genome-wide association studies.

#### 3.7.6 Number of alleles per locus pooled analysis

The pooled estimate for the number of alleles per locus was Na = 3.72 (95% CI: 3.13–4.30; 95% PI: 1.63–5.81), based on 10 studies. Heterogeneity remained high (I^2^ = 89.6%; Q = 84.69, p < 0.0001; τ^2^ = 0.766). An average of approximately four alleles per locus is consistent with moderate allelic richness, reflecting the biology of a crop that reproduces primarily through vegetative propagation. By comparison, outcrossing diploid species typically harbor 5 to 15 alleles per microsatellite locus, while predominantly selfing species may show only 2 to 4 (Hamrick and Godt, 1996). As indicated, only SSR-only analysis was feasible for Na given the biological limitations of SNP data.

#### 3.7.7 Publication bias assessment

Visual inspection of the funnel plot for He showed moderate asymmetry, with a slight deficit of studies reporting low He values with small standard errors. However, given the small number of studies (k = 12), formal assessment using Egger’s regression test was interpreted with caution. The test did not reach statistical significance (p = 0.12), suggesting that while some degree of publication bias cannot be entirely excluded, it is unlikely to substantially distort the pooled estimate (**Figure 12**).

**Figure 12:**
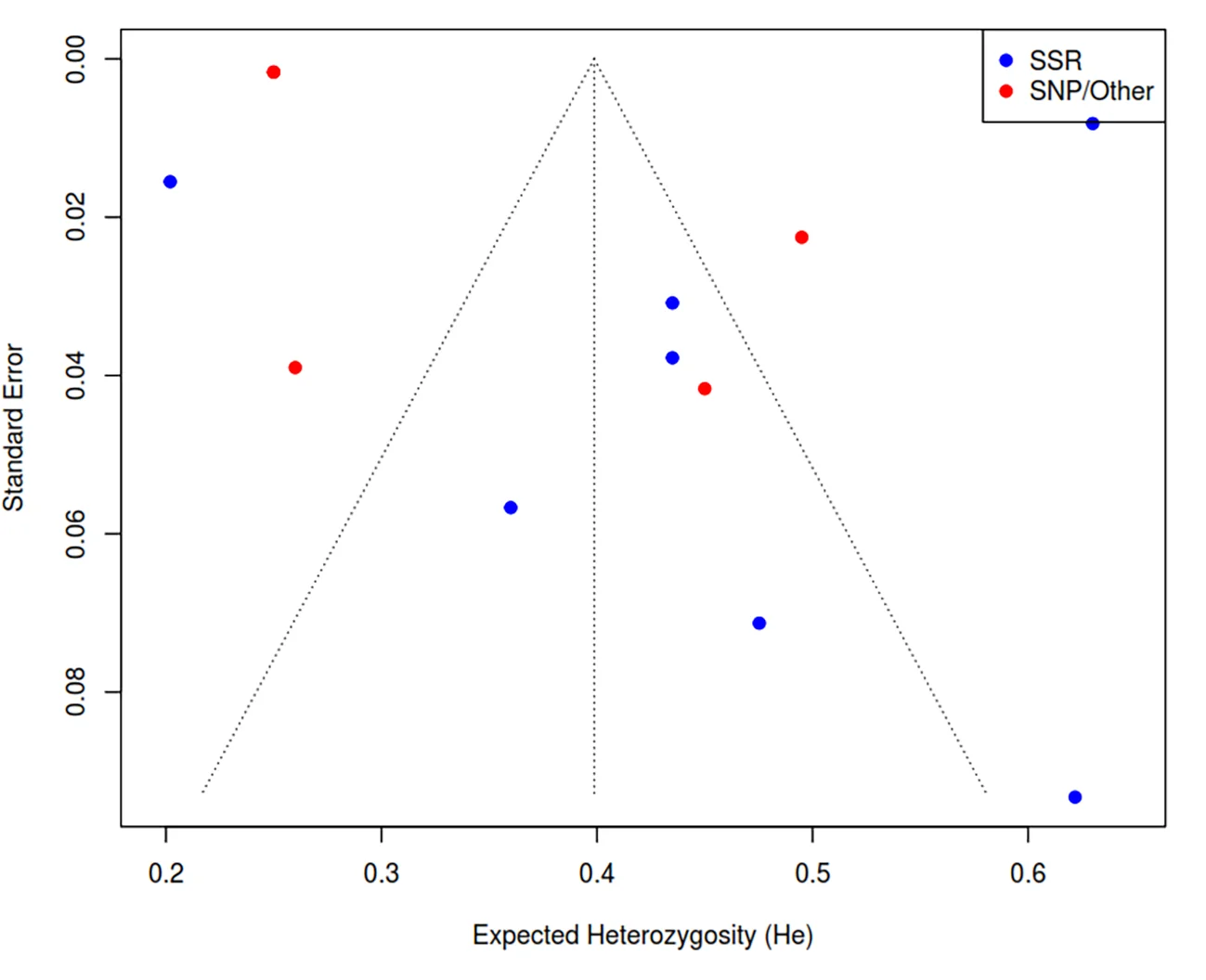
Funnel plot for the visual assessment of publication bias in the random-effects meta-analysis of expected heterozygosity (He) in African *Colocasia esculenta* (k = 12 studies). The x-axis represents individual study He estimates and the y-axis represents the standard error (inversely related to study precision). Vertical dashed lines indicate the pooled estimate (He = 0.40) and the 95% confidence interval; oblique lines represent the expected symmetric distribution under the null hypothesis of no publication bias. Asymmetry was formally tested using Egger's regression test (p = 0.12); no significant evidence of publication bias was detected.

#### 3.7.8 Sensitivity analyses

Two sensitivity analyses were conducted to assess the robustness of the pooled estimates. First, a leave-one-out analysis was performed by sequentially removing each study and re-running the meta-analysis. For He, the pooled estimate ranged from 0.37 to 0.43 across the 12 iterations, with no individual study exerting a disproportionate influence on the overall result. Similar stability was observed for PIC (range: 0.44–0.48) and Na (range: 3.65–3.82).

Second, the meta-analyses were repeated after excluding the four studies classified as Low quality (score < 5). When restricted to studies rated High or Medium quality (n = 40), the pooled He remained stable at 0.41 (95% CI: 0.33–0.49), essentially unchanged from the full analysis (He = 0.40; 95% CI: 0.32–0.48). Similar stability was observed for PIC (0.47 vs 0.46) and Na (3.78 vs 3.72). None of the four low-quality studies met our pre-specified exclusion criteria, and quality scores were used for sensitivity analysis rather than as exclusion criteria, consistent with our registered protocol (OSF DOI: 10.17605/OSF.IO/5S9PY). Together, these sensitivity analyses confirm that the pooled estimates are robust to both the exclusion of individual studies and the exclusion of studies with lower methodological quality (**Figure 13**).

**Figure 13:**
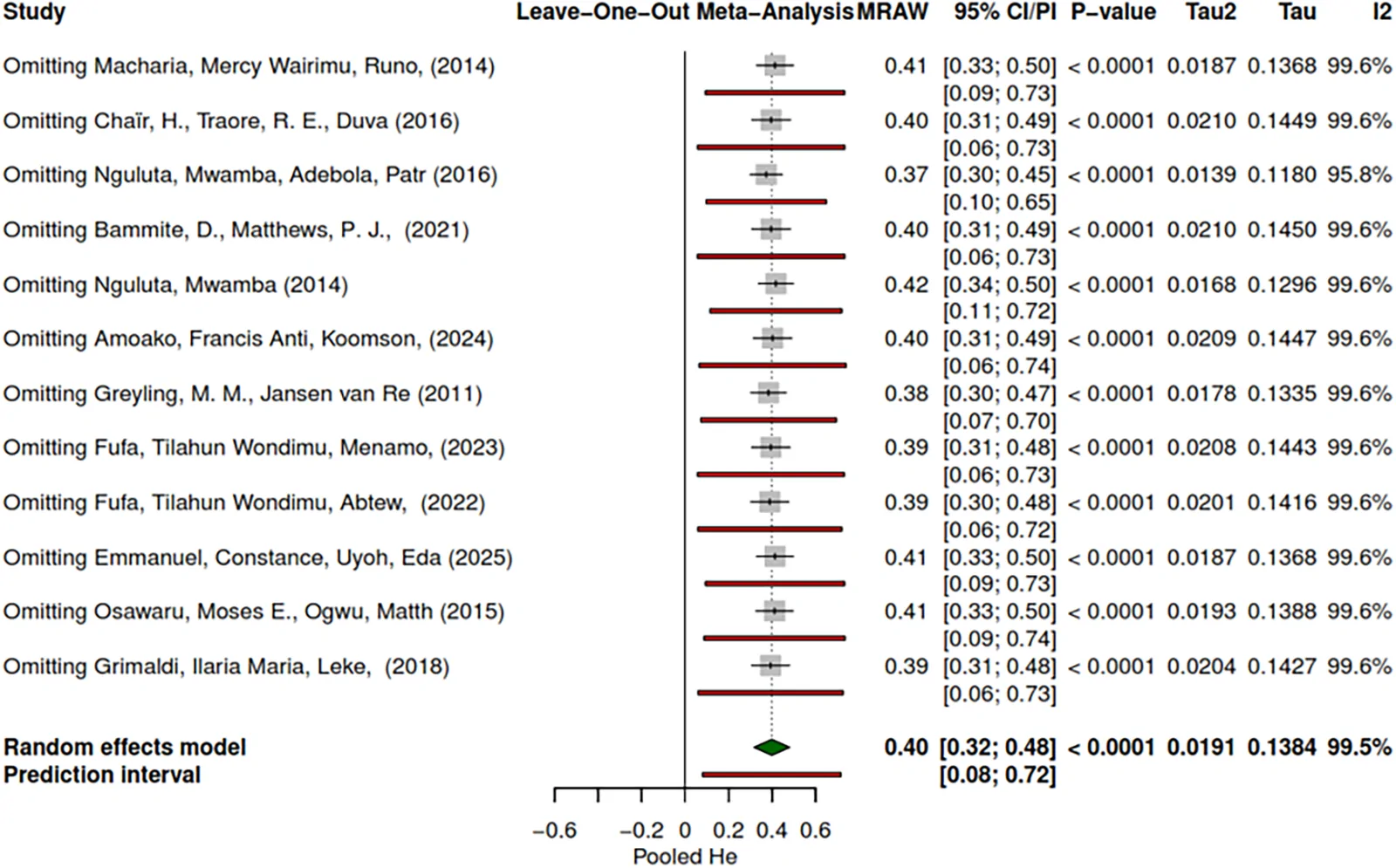
Leave-one-out sensitivity analysis for the random-effects pooled estimate of expected heterozygosity (He) in African *Colocasia esculenta* (k = 12 studies). Each row shows the recomputed pooled He estimate and 95% confidence interval after excluding the study named on the y-axis. The vertical dashed line indicates the original pooled estimate (He = 0.40) obtained with all 12 studies. The overall stability of the pooled estimate across all leave-one-out iterations (range: 0.38–0.42) indicates that no single study exerts disproportionate influence on the meta-analytical result, supporting the robustness of the pooled He estimate.

### 3.8 Summary of meta-analysis results

**Table 5** presents the consolidated results of the three overall meta-analyses performed in this review, while **Table 3** presents the marker-stratified results. Across all analyses, African taro shows moderate genetic diversity, with pooled estimates that are robust to methodological choices, sensitivity analyses, and quality-based exclusions.

**Table 5:** Summary of random-effects meta-analyses for molecular diversity metrics of *Colocasia esculenta* in Africa.

Variable	k	Pooled estimate [95% CI]	95% PI	I^2^ (%)	Q (p-value)	τ^2^
He	12	0.40 [0.32, 0.48]	[0.08, 0.72]	99.5	2348.87 (< 0.0001)	0.019
PIC	10	0.46 [0.38, 0.55]	[0.18, 0.75]	92.5	120.39 (< 0.0001)	0.015
Na	10	3.72 [3.13, 4.30]	[1.63, 5.81]	89.4	84.69 (< 0.0001)	0.766

k, number of studies; CI, confidence interval; PI, prediction interval; I^2^, proportion of total variation due to between-study heterogeneity; Q, Cochran’s Q statistic; τ^2^, between-study variance. All models fitted using restricted maximum likelihood (REML) estimation.

### 3.9 Complementarity of characterization approaches

Among the 44 original research articles, only three studies combined agromorphological and molecular approaches within the same experimental design. These integrated studies consistently demonstrated that morphological and molecular data capture partially overlapping but distinct dimensions of diversity. In Kenya, Macharia et al. (2014) showed that SSR-based clustering did not perfectly mirror morphological groupings, with some genetically similar accessions displaying divergent phenotypic profiles due to environmental plasticity. This finding underscores the complementary value of combining multiple characterization approaches, a recommendation that has been echoed in reviews of genetic resource management for underutilised crops (Bioversity International, 2010).

The near-complete absence of studies integrating all three characterization dimensions agromorphological, biochemical, and molecular represents a notable gap. A holistic approach combining phenotypic evaluation, nutritional profiling, and molecular fingerprinting would provide the most comprehensive assessment of taro genetic resources and would be particularly valuable for breeding programs seeking to simultaneously improve yield, nutritional quality, and adaptive traits.

## 4 Discussion

This study represents, to our knowledge, the first systematic review with quantitative meta-analysis specifically dedicated to the characterization of *Colocasia esculenta* across the African continent. By integrating agromorphological, biochemical, and molecular data from 44 original research articles published between 2010 and 2025, this work provides a consolidated picture of the current state of knowledge on taro genetic resources in Africa. The discussion that follows examines the key findings in relation to the existing literature, identifies critical research gaps, and proposes directions for future investigation.

### 4.1 A moderate but meaningful genetic diversity in African taro

The pooled expected heterozygosity of 0.40, derived from 12 independent molecular studies, positions African taro in the moderate diversity range. This value, while lower than what is typically observed in sexually reproducing crop species, is not surprising for a predominantly vegetatively propagated crop. In cassava, another clonally propagated staple of tropical agriculture, meta-analytical estimates of He generally fall between 0.35 and 0.55 across African populations (Kawuki et al., 2013; Rabbi et al., 2015). Yam species, which combine vegetative and occasional sexual reproduction, tend to show slightly higher diversity with He values of 0.40 to 0.65 depending on the species and region (Scarcelli et al., 2019; Agre et al., 2019). The taro He value reported here is thus consistent with the expected diversity profile for a crop of its reproductive biology.

What makes this finding noteworthy, however, is the implication that African taro populations have not been reduced to a handful of genetically uniform clones despite centuries of vegetative propagation. This apparent robustness of diversity contrasts with classical population genetics predictions, which suggest that predominantly clonally propagated species should exhibit reduced within-population diversity relative to sexually reproducing species, as vegetative reproduction reduces effective recombination and can lead to allele fixation or loss through clonal drift and selection on entire multilocus genotypes (Balloux et al., 2003; De Meeus et al., 2007). Several mechanisms may account for this retained diversity. First, the occasional production of viable seeds in taro, though rarely exploited by farmers, can introduce new recombinant genotypes into local populations (Matthews, 2004; Kreike et al., 2004). Second, the traditional farming systems prevalent across much of Africa characterized by polyculture, farmer-to-farmer seed exchange, and the maintenance of multiple named varieties tend to preserve genetic variation more effectively than industrialized monoculture systems (Jarvis et al., 2008). In this context, African farmers themselves function as active custodians of taro diversity: their selection of morphotypes on the basis of culinary preferences, agronomic performance, or cultural significance, together with the intergenerational transmission of named landraces, constitutes a de facto form of *in situ* conservation that may maintain phenotypic and genetic variation which formal molecular surveys, focused on a small number of neutral loci, could easily overlook. Third, the wide geographic distribution of taro cultivation across ecologically diverse African environments has likely promoted local adaptation and the maintenance of region-specific alleles through isolation by distance. Fourth, the polyploid nature of many African taro accessions (2n = 2x, 3x, 4x) can buffer against allele loss and inflate observed heterozygosity through fixed heterozygosity at homoeologous loci, providing an additional structural mechanism for the maintenance of high diversity levels in clonal populations. Together, these processes support the emerging view that vegetatively propagated crops can maintain substantial genetic diversity when managed within traditional agricultural systems, underscoring the importance of on-farm conservation strategies for preserving the evolutionary potential of African taro. More broadly, these mechanisms illustrate a general principle recognized in the plant genetic resources literature: the very status of taro as an “orphan crop” — a species that has received limited attention from formal breeding programs and modern intensive agricultural practices — has paradoxically served to preserve its genetic diversity, since the domestication bottlenecks, elite variety displacement, and cultivar homogenization typically associated with commodity crops have largely not occurred for African taro (Padulosi et al., 2013; Dawson et al., 2019).

The pooled PIC of 0.46 further supports the picture of moderate diversity. This value sits near the boundary between the moderately and highly informative categories defined by Botstein et al. (1980), indicating that the marker systems employed across the reviewed studies have been reasonably effective at detecting polymorphism. Nevertheless, the finding also suggests room for improvement. As taro genomic resources continue to develop including the availability of reference genome assemblies and high-density SNP platforms future studies should be able to capture finer-scale genetic variation that may have been missed by the microsatellite panels used in most existing work.

The average of 3.72 alleles per locus, while modest in absolute terms, is within the expected range for microsatellite markers in vegetatively propagated crops. For perspective, Hamrick and Godt (1996), in their seminal review of allozyme diversity across plant species, reported mean allelic richness values of 1.5 to 2.5 for predominantly selfing species and 3.0 to 5.0 for species with mixed or outcrossing mating systems. The value observed here is thus consistent with the intermediate reproductive strategy of taro, which combines predominant clonal propagation with occasional outcrossing.

### 4.2 The transition from phenotypic to genomic characterization

One of the more striking patterns to emerge from this review is the clear temporal shift in the methodological approaches employed for taro characterization in Africa. During the early part of the study period (2010–2015), agromorphological assessment using IPGRI descriptors was the predominant approach, reflecting its relative simplicity, low cost, and suitability for resource-limited settings. These early studies provided essential baseline data on phenotypic variation within national taro collections and identified morphological traits with potential value for breeding, such as corm shape, plant height, and leaf dimensions.

From 2016 onward, molecular characterization became increasingly common, initially through microsatellite genotyping and more recently through SNP-based approaches including genotyping-by-sequencing. This transition mirrors a broader trend across crop genetic resource research in Africa, driven by declining sequencing costs, growing bioinformatics capacity, and the establishment of shared genomic platforms at regional centers of excellence (Varshney et al., 2021). In our dataset, SSR markers accounted for 58.8% of all molecular marker entries, while SNP and GBS-based approaches accounted for 29.4%, a proportion that is likely to increase rapidly in the coming years.

This methodological evolution has important consequences for the interpretation of diversity estimates. SSR markers, with their high mutation rates and multi-allelic nature, tend to reveal higher levels of polymorphism per locus than SNP markers, which are typically bi-allelic. The subgroup analysis in our meta-analysis confirmed this pattern, with SSR-based studies yielding a higher pooled He (0.45) than studies using other marker systems (0.31). This marker-dependent bias is a well-documented phenomenon in comparative genetics and must be considered when making cross-study comparisons (Kalia et al., 2011; Semagn et al., 2006). It also partly explains the very high heterogeneity (I^2^ = 99.5%) observed in our pooled He estimate, as the mixing of fundamentally different marker technologies inflates between-study variance.

Despite the growing prevalence of molecular tools, our review revealed that agromorphological characterization remains indispensable, particularly in applied breeding contexts. Several of the most informative studies in our dataset combined both approaches. For instance, Debela et al. (2025) demonstrated through AMMI and GGE biplot analyses that genotype-by-environment interactions significantly affected taro yield performance across six environments in southwestern Ethiopia a dimension of variation that molecular markers alone cannot capture. Similarly, the agromorphological studies consistently identified corm shape, plant height, and leaf dimensions as the traits with the greatest discriminating power, information directly relevant to variety selection and farmer participatory evaluation.

### 4.3 Geographic imbalances and blind spots

The geographic distribution of taro characterization studies in Africa is markedly uneven. Nigeria alone contributed 16 of the 44 original studies (including recent large-scale genetic diversity studies of Nigerian taro cultivars; Oladimeji et al., 2024), followed by Kenya (9), South Africa (9), and Ethiopia (4). Together, these four countries accounted for over 86% of all original research identified in this review. West Africa, as a region, contributed 45.5% of all studies, reflecting both the crop’s dietary importance in the region and the presence of active research programs at institutions such as the National Root Crops Research Institute in Umudike and the Council for Scientific and Industrial Research in Ghana.

The concentration of research in these four countries reflects several converging factors beyond mere research capacity. First, these countries host established national agricultural research systems (NARS) with sustained programs on taro and related root crops: the National Root Crops Research Institute (NRCRI) at Umudike in Nigeria, the Kenya Agricultural and Livestock Research Organization (KALRO), the Agricultural Research Council (ARC) in South Africa, and the Ethiopian Institute of Agricultural Research (EIAR) have all maintained taro breeding and characterization activities over the past two decades. Second, the presence of CGIAR-affiliated programs—notably the International Institute of Tropical Agriculture (IITA) with hubs in Ibadan and Nairobi—has generated collaborative networks that facilitate research and publication in these countries. Third, international networks such as the International Network for Edible Aroids (INEA) and regional partnerships have historically prioritized these countries in taro-related activities.

Historical patterns of research investment and colonial legacy also play a role in the observed geographic distribution. Countries with longer traditions of anglophone academic engagement, or with well-established public research institutions dating back to the colonial and post-colonial periods, tend to be better represented in international scientific literature. The relative visibility of taro research in some regions may also depend on the crop’s cultural and dietary significance, with countries where taro is a staple food receiving more sustained scientific attention than countries where it is a minor crop.

By contrast, Central Africa—encompassing major taro-producing countries such as Cameroon, the Democratic Republic of Congo, and the Central African Republic—was represented by only a single study in our review. North Africa was entirely absent from the dataset. This geographic asymmetry represents a significant knowledge gap, particularly for Cameroon, which ranks among the top taro producers on the continent according to FAO production statistics (FAOSTAT, 2023). The island nations of Madagascar, Comoros, and Mauritius, where taro also holds cultural and nutritional significance, were similarly unrepresented. The underrepresentation of Central Africa likely reflects a combination of language restrictions, limited institutional research capacity specifically for taro, and less-developed international collaboration networks in these regions.

These gaps have tangible consequences for the comprehensiveness of diversity assessments. Taro germplasm from undersampled regions may harbor unique adaptive traits—drought tolerance, disease resistance, nutritional qualities—that have not yet been documented or incorporated into breeding programs. The concentration of research in a few countries also creates a sampling bias that limits the generalizability of our meta-analytical findings. We cannot, for example, confidently assert that the pooled He of 0.40 accurately represents the genetic diversity of taro across the entire continent when major production zones remain unstudied. Furthermore, the observed geographic pattern may partly reflect biases in scientific visibility rather than in the actual distribution of taro research, since studies published in local French- or Portuguese-language journals would not have been captured by our English-only search strategy.

Addressing these gaps will require deliberate investment in research capacity and infrastructure in underrepresented regions, together with support for scientific publishing in African journals and in multiple languages. Multi-country collaborative projects, such as those facilitated by the International Network for Edible Aroids or the CGIAR system, offer a promising framework for expanding the geographic scope of taro characterization in Africa. The development of shared genotyping platforms, standardized phenotyping protocols, and multilingual open-access repositories would further enable meaningful cross-country comparisons and reduce the current visibility gap between anglophone and francophone taro research.

### 4.4 Sources of heterogeneity in the meta-analysis

All three meta-analyses conducted in this review exhibited high to very high between-study heterogeneity, with I^2^ values exceeding 89% for each variable. While such values might initially seem alarming, they are consistent with the methodological and biological heterogeneity inherent in the pooled datasets and do not, in themselves, invalidate the meta-analytical approach. Rather, they call for a careful examination of the sources of variation, which our subgroup, stratified, and meta-regression analyses were designed to address. Three principal sources of heterogeneity merit discussion. First, the mixing of different marker technologies each with distinct mutation rates, allelic spectra, and resolving power inevitably inflates between-study variance. The marker-stratified analyses reported in this point, showing that SSR-only and SNP/GBS-only pooled He estimates differ by approximately 0.11 units (0.45 versus 0.34), and that between-study heterogeneity for PIC was substantially reduced when restricted to SSR-based studies alone (I^2^ dropping from 92.5% to 74.5%). Second, differences in sample composition played a role: studies analyzing broad geographic panels encompassing multiple countries naturally captured more allelic diversity than those focused on local collections from a single district. Third, variation in sample size from fewer than 20 accessions in some studies to over 400 in others affected the precision of individual He estimates and contributed to the observed dispersion. The high heterogeneity underscores the importance of interpreting the pooled estimates as indicative summary measures rather than precise universal parameters. The prediction intervals, which were wider than the confidence intervals, provide a more realistic reflection of the expected range of diversity values in future individual studies. For He, the 95% prediction interval extended from 0.08 to 0.72, encompassing nearly the full range of values observed across the individual studies. Similar prediction intervals were obtained for PIC (0.18–0.75) and Na (1.63–5.81), each substantially wider than the corresponding confidence intervals. These prediction intervals should be understood as the more realistic reference range for anticipated diversity values in future primary studies of African taro.

It is important to emphasize that the prediction intervals reported here should be considered as the primary reference range for anticipating diversity values in future studies of African taro, rather than the narrower confidence intervals traditionally reported in the literature. Whereas the confidence interval quantifies the uncertainty around the pooled mean given the observed studies, the prediction interval accounts for the true between-study variability and reflects the range within which the diversity value of a future comparable study is likely to fall (Hedges and Pigott, 2004; Higgins et al., 2009; IntHout et al., 2016). In our data, the width of the prediction intervals (spanning approximately 0.64 units for He, 0.57 units for PIC, and 4.18 units for Na) is such that they cover nearly the full range of individual study estimates in each meta-analysis. This has an important practical implication: pooled estimates derived from the overall (marker-mixed) meta-analyses are of limited value for direct cross-study comparisons or for benchmarking a single new study against a universal expected value. Researchers designing new characterization studies or interpreting their results against the meta-analytical literature should therefore rely preferentially on the marker-stratified pooled estimates presented in **Table 3** (SSR-only, SNP/GBS-only) rather than on the overall pooled estimates, and should interpret any comparison against the prediction interval of the appropriate marker-specific subset rather than the narrower confidence interval of the overall pool.

The meta-regression analyses offer a complementary lens on the sources of heterogeneity. Contrary to the intuitive expectation that marker type, number of loci, sample size, or geographic region would explain a measurable proportion of the between-study variance, none of these four moderators reached statistical significance in the univariate meta-regression models, and the proportion of heterogeneity explained (R^2^) was estimated at zero for each moderator. This apparent inconsistency with the marker-stratified analyses reported in where SSR and SNP/GBS-based He estimates differed by 0.11 units, is best explained by two convergent factors. First, the small number of studies contributing to the He meta-analysis (k = 12) severely constrains the statistical power of meta-regression, as standard recommendations require at least ten studies per moderator (Borenstein et al., 2009). The direction and magnitude of the marker effect identified in the stratified analyses are thus likely real, but the current evidence base is insufficient to detect them at conventional significance thresholds. Second, and perhaps more importantly, the residual heterogeneity remained very high in all meta-regression models (I^2^ > 99%), suggesting that variability between studies is driven by factors that our moderators could not capture. These unobserved factors may include specific SSR primer panels employed, DNA extraction and quantification protocols, allele-calling algorithms, and sample selection strategies, none of which are consistently reported in the primary literature.

This inability to identify significant moderators is itself an informative finding rather than a limitation of our approach. It highlights the need for improved reporting standards in molecular characterization studies of African taro. Systematic disclosure of methodological details primer sequences, extraction methods, allele-calling parameters, and sampling strategies would substantially enhance the potential of future meta-analyses to identify and quantify the sources of between-study variability. In the meantime, the marker-stratified analyses provide more directly interpretable diversity estimates for each marker technology, and we recommend that future comparisons of African taro diversity across regions or time periods rely on marker-specific pooled estimates rather than the overall pooled value, at least until a larger evidence base allows more powerful meta-regression modelling.

Importantly, the non-significant meta-regression results should not be interpreted as evidence that the tested moderators (marker type, number of loci, number of accessions, and African region) are unimportant determinants of the observed diversity estimates. Rather, with only 12 studies for He and even fewer for PIC and Na, the meta-regression models were substantially underpowered to detect even moderate effects. The direction of the marker-related effect identified in the stratified analyses (0.11-unit difference between SSR and SNP/GBS pooled He estimates) is biologically plausible and consistent with the theoretical expectation that codominant microsatellite markers should yield higher He values than biallelic SNPs. Similarly, the differences observed across African sub-regions in the descriptive analyses suggest genuine geographic patterns that may become statistically detectable in future updates of this review. As the body of primary studies grows and as reporting standards improve, future meta-regression analyses conducted with a larger evidence base will likely be able to quantify these effects with greater precision. Our current findings should therefore be interpreted as a baseline that identifies both the diversity signal detectable in the existing literature and the methodological improvements needed to sharpen future syntheses.

From a methodological standpoint, these findings reinforce the value of conducting subgroup, stratified, and meta-regression analyses in systematic reviews of genetic diversity data. The approach adopted in this study stratifying by marker type, geographic region, and publication period follows the analytical framework employed by Mwesigwa et al. (2024) in their meta-analysis of Plasmodium falciparum genetic diversity in sub-Saharan Africa, and by van de Wouw et al. (2010) in their assessment of temporal trends in crop genetic diversity. These precedents support the validity of pooling diversity metrics across studies while acknowledging the limitations imposed by methodological heterogeneity.

### 4.5 The missing dimension: biochemical characterization

Among the three characterization approaches examined in this review, biochemical profiling emerged as the most neglected. Only five original studies provided quantitative data on the nutritional or anti-nutritional composition of African taro accessions, representing barely 11% of the included literature. This scarcity is striking given the growing global interest in the nutritional properties of underutilised crops and the potential of taro as a source of resistant starch, hypoallergenic protein, and essential minerals (Aditika et al., 2022; Temesgen and Retta, 2015). The absence of a quantitative meta-analysis for biochemical parameters, despite the availability of five original studies reporting overlapping metrics, reflects a fundamental methodological limitation of the available evidence rather than a limitation of our review approach. The five biochemical studies employed different analytical methods (varying enzymatic and gravimetric protocols for starch, distinct Kjeldahl and combustion approaches for protein with different nitrogen-to-protein conversion factors, and differing titrimetric, spectrophotometric, and enzymatic methods for oxalates), reported results on inconsistent bases (fresh vs. dry weight; per 100 g vs. percentage), and frequently omitted measures of dispersion required for meta-analytical pooling. Attempting to pool these heterogeneous estimates would produce a statistically defined but biologically uninterpretable summary. This methodological fragmentation is itself a diagnostic finding: it reveals that biochemical characterization of African taro suffers not only from a low number of studies but also from a lack of consensus on standardized analytical protocols.

The limited biochemical data identified in this review, though insufficient for meaningful meta-analysis, nonetheless reveal considerable inter-accession variability in key nutritional parameters. Starch content ranged from 15% to 28% on a dry weight basis, while crude protein varied from 1.5% to 7%. Oxalate content, a major determinant of palatability and an anti-nutritional concern, showed similarly wide variation, suggesting that targeted selection for low-oxalate genotypes is feasible if adequate screening is performed.

The gap in biochemical characterization has practical implications. Breeding programs seeking to develop nutritionally superior taro varieties higher protein, lower oxalates, enhanced mineral density lack the comprehensive baseline data needed to identify promising parental material. Future research should therefore pursue two complementary priorities. First, the systematic biochemical profiling of the diverse germplasm collections already assembled across African genebanks should be prioritized, using standardized analytical protocols that enable cross-study comparisons. The harmonization of biochemical assay methods across research groups ideally through the adoption of internationally recognized standards such as those recommended by AOAC International should be considered a prerequisite for enabling future quantitative synthesis in this domain. Second, the integration of biochemical data with molecular fingerprinting through genome-wide association studies would represent a particularly powerful approach for identifying genomic regions controlling nutritional traits and accelerating marker-assisted selection for improved nutritional quality.

Beyond these methodological considerations, the scarcity of biochemical studies (k = 5) is also insufficient for meaningful meta-regression and represents a substantive gap at a time when biochemical characterization has become strategically important for African food systems. *Colocasia esculenta* is a recognized source of resistant starch, a functional food component associated with improved glycemic control and colonic health, and offers a favorable profile of essential minerals including potassium, magnesium, and iron. In the context of ongoing continental initiatives on biofortification, nutrient-sensitive agriculture, and dietary diversification for food and nutrition security, the near-absence of standardized biochemical data for African taro germplasm significantly limits the ability of breeding programs to select for nutritional quality traits alongside agronomic performance. This gap is particularly concerning because complementary morphological and molecular characterization data are increasingly available, meaning that biochemical profiling has become the rate-limiting step in comprehensive genetic resource evaluation. Future research on African taro should therefore treat biochemical profiling as a research priority rather than an afterthought, and national and international funding agencies should consider targeted calls for integrated phenotypic–genotypic–nutritional characterization of neglected and underutilised crops. Such integrated approaches, combined with the standardized analytical protocols discussed above, would substantially accelerate the transition of African taro from a locally important subsistence crop toward a fully characterized genetic resource capable of contributing to continental food and nutrition security.

### 4.6 Towards integrated multi-dimensional characterization

Perhaps the most important finding of this review, from a strategic perspective, is the almost complete lack of studies integrating all three characterization dimensions within a single experimental framework. Of the 44 original studies, only three combined agromorphological and molecular approaches; none incorporated biochemical profiling alongside the other two. This compartmentalization of research efforts limits the ability to draw holistic conclusions about taro genetic resources and hampers the development of marker-assisted selection strategies that account for both agronomic performance and nutritional value.

The complementary nature of the three approaches is well established in principle. Agromorphological characterization captures phenotypic variation directly relevant to farmer preferences and market requirements, but it is subject to environmental plasticity and genotype-by-environment interactions. Molecular characterization provides genotype-level resolution that is independent of environmental effects, enabling unambiguous identification of genetic relationships and population structure. Biochemical characterization adds the nutritional dimension, which is increasingly central to crop improvement strategies aligned with public health objectives (Bouis and Saltzman, 2017).

Integrated studies that simultaneously deploy all three approaches on the same germplasm collections would yield several specific benefits. They would enable the identification of molecular markers associated with agronomically important traits through association mapping. They would reveal the extent to which morphological clustering corresponds to genetic relatedness or diverges from it due to convergent adaptation. And they would facilitate the construction of comprehensive core collections that capture maximum diversity across phenotypic, genetic, and nutritional dimensions.

We therefore recommend that future taro characterization efforts in Africa adopt a multi-dimensional design as a standard practice. The declining costs of molecular genotyping and the increasing availability of standardized biochemical assay kits make such integrated approaches increasingly feasible, even in resource-constrained research environments.

### 4.7 Implications for breeding and conservation

The moderate genetic diversity documented in this review carries important implications for both taro improvement and conservation strategies in Africa. On the breeding front, the existence of meaningful phenotypic and genetic variation among African taro accessions provides a foundation for selection and hybridization. The identification of high-yielding genotypes such as JD/Ce23 in Ethiopia, with mean yields exceeding 37 tonnes per hectare (Debela et al., 2025), and the documentation of substantial variation in corm weight, maturity, and eating quality across West African collections (Boampong et al., 2018) suggest that considerable genetic gain is achievable through systematic breeding. The relatively high heritability estimates reported for traits such as petiole length and plant height (Tewodros, 2013) further indicate that these characters should respond predictably to selection.

On the conservation front, the uneven geographic coverage revealed by this review raises concerns about the representativeness of existing *ex situ* collections. If the taro germplasm held in African genebanks reflects the same research biases documented here with strong representation from Nigeria, Kenya, South Africa, and Ethiopia but weak coverage of Central Africa, North Africa, and island nations then important adaptive variation may remain uncollected and at risk of genetic erosion. Given the increasing pressures of land use change, urbanization, and climate variability on traditional farming systems across Africa, targeted collection missions in underrepresented regions should be treated as a matter of urgency.

The development of core collections subsets of germplasm that capture the maximum genetic diversity with a minimum number of accessions represents a practical strategy for making large taro collections more manageable and accessible to breeders. The molecular diversity data synthesized in this review can inform the design of such core collections, while agromorphological and biochemical data can guide their refinement for specific breeding objectives.

### 4.8 Limitations of this review

Several limitations should be considered when interpreting the findings of this review. First, the literature search was restricted to English-language publications in five electronic databases, which may have excluded relevant studies published in French, Portuguese, or other languages. This is a particularly important limitation for a systematic review focused on Africa, given that many major taro-growing African countries including Côte d’Ivoire, Cameroon, Benin, Burkina Faso, Togo, Madagascar, and the Democratic Republic of Congo have French as their primary academic and administrative language, while additional taro-producing regions publish primarily in Portuguese.

However, the practical impact of this restriction must be assessed with nuance. In most francophone African countries, the majority of peer-reviewed scientific publications on agricultural and plant-genetic topics are nonetheless written in English, reflecting the international norms of scientific communication and the incentive structures of academic evaluation systems that reward publication in English-language journals indexed in international databases. As a result, our search may still have captured a substantial fraction of the primary research output from francophone Africa, particularly studies published in international or high-impact regional journals. Nevertheless, an unknown but potentially non-negligible fraction of scientific output including theses, national bulletins, technical reports, and articles in local French- or Portuguese-language journals would have been missed. Drawing on the methodological literature on language bias in systematic reviews, English-language restriction typically leads to the omission of 6-12% of relevant studies for topics related to Africa (Morrison et al., 2012), corresponding in our case to approximately 3–5 additional studies potentially missed. While empirical evidence indicates that such omissions rarely alter pooled quantitative estimates substantially (Nussbaumer-Streit et al., 2020), the qualitative interpretation of geographic gaps — particularly for Central Africa — is more affected. The underrepresentation of Central Africa in our dataset may therefore partly reflect this language restriction, representing a partial confound with the geographic capacity gaps. Future updates of this systematic review should include French-language databases such as HAL (Hyper Articles en Ligne), Cairn.info, and BASE (Bielefeld Academic Search Engine), and Portuguese-language sources such as SciELO, to provide a more complete picture of the African taro research landscape and to disentangle language-related biases from genuine research-capacity gaps.

Similarly, the absence of Scopus and Web of Science from the search, due to lack of institutional access, may have resulted in the omission of some studies indexed exclusively in those databases, although OpenAlex was included as a mitigating measure to broaden coverage of open-indexed literature.

Second, the quantitative data extracted from the original studies were sometimes reported in heterogeneous formats—as means with standard deviations, as ranges without measures of dispersion, or embedded in figures rather than tables. In such cases, approximations were necessary, including the estimation of standard deviations from reported ranges, which introduces an additional source of uncertainty into the meta-analytical estimates.

Third, the very high heterogeneity observed across all three meta-analyses limits the precision of the pooled estimates and their generalizability to individual populations or marker systems. While subgroup analyses, marker-stratified analyses, meta-regression, and sensitivity analyses helped to contextualize this heterogeneity, they could not fully resolve it, reflecting the fundamental methodological diversity of the underlying studies. Importantly, however, our quality-based sensitivity analyses demonstrated that the pooled estimates are robust to the exclusion of the four low-quality studies, with only marginal changes in the pooled He (0.40 vs 0.41), PIC (0.46 vs 0.47), and Na (3.72 vs 3.78) values. This robustness supports the validity of our main findings and suggests that the inclusion of these lower-quality studies did not substantially bias the meta-analytical estimates. However, this observation should not be interpreted as an endorsement of low methodological quality in individual studies; rather, it reflects the inherent statistical resilience of random-effects meta-analytical estimates to the inclusion of a small proportion of lower-quality studies within a larger evidence base. Future primary studies should aim for the highest methodological standards to strengthen the evidence available for future syntheses.

Finally, the small number of studies available for certain variables particularly biochemical parameters and some molecular metrics precluded formal meta-analysis for these outcomes and limited the statistical power of the analyses that were performed, including meta-regression models where all four moderators tested (marker type, number of loci, number of accessions, and region) failed to reach statistical significance despite the direction of effects observed in the stratified analyses. As the body of taro characterization literature in Africa continues to grow, future updates of this systematic review should be able to address several of these limitations, particularly if standardized reporting practices are adopted and multilingual databases are systematically consulted. A striking cross-cutting finding of this systematic review is the complete absence of integrated tri-dimensional characterization studies. Of the 44 original research articles reviewed, none simultaneously combined agromorphological, biochemical, and molecular approaches on the same set of accessions: three studies were classified as “mixed” but integrated only two dimensions (agromorphological and molecular), leaving 41 studies focused on a single characterization approach. This 0/44 gap is methodologically significant because it prevents the direct linking of morphological traits, nutritional quality parameters, and molecular markers within a common experimental framework. Such linkage is a prerequisite for genotype-phenotype-nutrition association studies, marker-assisted selection for nutritional traits, and the development of nutrient-dense taro varieties. The absence of tri-dimensional studies represents both a diagnostic finding about the fragmented state of taro research in Africa and a clear research priority for the coming decade.

## 5 Conclusion

This systematic review examined 64 studies including 44 original research articles and 20 reviews published between 2010 and 2025 on the agromorphological, biochemical, and molecular characterization of *Colocasia esculenta* across the African continent. Through a quantitative meta-analysis of molecular diversity metrics, pooled estimates of expected heterozygosity, polymorphism information content, and number of alleles per locus were obtained, collectively pointing to a moderate level of genetic diversity within African taro germplasm. These values, while reflective of a crop that relies predominantly on vegetative propagation, indicate that a meaningful reservoir of allelic variation persists across the continent and remains available for exploitation in breeding programs. Sensitivity analyses confirmed that these pooled estimates are robust to the exclusion of any individual study and to the exclusion of studies of lower methodological quality, supporting the reliability of our meta-analytical findings.

Several important patterns emerged from the synthesis. The research landscape has undergone a clear methodological shift over the past fifteen years, moving from phenotype-based assessments using standardized descriptors toward molecular approaches anchored in microsatellite and, more recently, SNP genotyping. This transition has enhanced the resolution of diversity analyses but has also introduced marker-dependent biases that must be acknowledged when comparing results across studies. The geographic coverage of existing research is strikingly uneven, with Nigeria, Kenya, South Africa, and Ethiopia contributing the vast majority of published work, while Central and North Africa remain virtually unexplored. Biochemical characterization, despite its direct relevance to nutrition and food security, constituted barely one-tenth of the literature reviewed.

On the basis of these findings, five recommendations are proposed for future research. First, targeted germplasm collection and characterization missions should be prioritized in underrepresented regions, particularly Central Africa, North Africa, where locally adapted taro diversity is likely present but undocumented. Second, future studies should adopt integrated designs that combine agromorphological, biochemical, and molecular approaches within a single experimental framework. A striking finding of this review is that not a single one of the 44 original studies (0/44) simultaneously addressed all three characterization dimensions on the same set of accessions, representing a major methodological gap that limits our ability to identify the genotype-phenotype-nutrition links needed to accelerate the improvement of African taro. Integrated tri-dimensional studies would enable a more complete assessment of genetic resources and the identification of genomic regions governing traits of agronomic and nutritional interest. Third, the transition toward high-density SNP and sequencing-based markers should be accelerated to facilitate genome-wide association studies and genomic selection in taro. Fourth, systematic biochemical profiling of existing germplasm collections should be undertaken using standardized protocols, with particular attention to anti-nutritional factors such as oxalates and to micronutrient density. Fifth, the development of genetically informed core collections, drawing on the diversity data synthesized in this review, would make the wealth of African taro germplasm more accessible to breeders and researchers. Two cross-cutting priorities also emerge: the improvement of methodological reporting standards in primary studies including systematic disclosure of primer panels, extraction protocols, and sampling strategies would substantially enhance the potential of future meta-analyses to explain the sources of between-study heterogeneity; and the inclusion of French- and Portuguese-language databases in future updates of this systematic review would reduce the geographic visibility gap between anglophone and francophone taro research on the continent.

Taro remains one of Africa’s most undervalued food crops. Its resilience to marginal growing conditions, its nutritional versatility, and the genetic diversity documented in this review collectively argue for a more sustained and coordinated research effort. The evidence assembled here should serve as both a baseline and a call to action for the scientific community engaged in the conservation and improvement of this crop across the continent.

## Data Availability

The original contributions presented in the study are included in the article/**Supplementary Material**. Further inquiries can be directed to the corresponding author.
