# Supplementary material for: Mapping the characterization landscape of Colocasia esculenta (L.) in Africa: gaps, trends, and priorities - a systematic review with meta-analysis
Source: Front Plant Sci. 2026 Sep 21;17:1930197. doi: 10.3389/fpls.2026.1930197 (PMC13635379; doi:10.3389/fpls.2026.1930197)
Supplement: Supplementary file 1 [file SupplementaryFile1.docx]

PRISMA 2020 Checklist

Supplementary Material S1 — PRISMA 2020 Checklist for the systematic review: "Mapping the characterization landscape of *Colocasia esculenta* in Africa: gaps, trends, and priorities from a systematic review with meta-analysis"

From: Page MJ, McKenzie JE, Bossuyt PM, Boutron I, Hoffmann TC, Mulrow CD, et al. The PRISMA 2020 statement: an updated guideline for reporting systematic reviews. BMJ 2021;372: n71. doi: 10.1136/bmj. n71

For more information, visit: <http://www.prisma-statement.org/>

| **Section** | **Item** | **Checklist Item** | **Location in Manuscript** | **Notes / Details** |
| --- | --- | --- | --- | --- |
| **TITLE** | | | | |
| Title | 1 | Identify the report as a systematic review. | Title page | Title includes 'systematic review with meta-analysis'. |
| **ABSTRACT** | | | | |
| Abstract | 2 | See the PRISMA 2020 for Abstracts checklist. | Abstract section | Structured abstract with Background, Objectives, Methods, Results, Conclusions (300 words). |
| **INTRODUCTION** | | | | |
| Introduction | 3 | Describe the rationale for the review in the context of existing knowledge. | Section 1, paragraphs 1-6 | Importance of taro for food security in Africa; existing characterization approaches; absence of continental-scale synthesis. |
| Introduction | 4 | Provide an explicit statement of the objective(s) or question(s) the review addresses. | Section 1, paragraph 7 | Three explicit objectives stated: (i) inventory studies, (ii) quantify pooled genetic diversity, (iii) identify gaps and recommendations. |
| METHODS | | | | |
| Methods | 5 | Specify the inclusion and exclusion criteria for the review and how studies were grouped for the syntheses. | Section 2.1 | PECO framework (Table 1); 6 inclusion criteria (I1-I6) and 7 exclusion criteria (E1-E7); studies grouped by characterization type (agromorphological, biochemical, molecular). |
| Methods | 6 | Specify all databases, registers, websites, organisations, reference lists and other sources searched or consulted. | Section 2.2 | Five databases: PubMed, Google Scholar, AGRIS, AJOL, OpenAlex. Search conducted March-April 2026. Scopus and Web of Science not available due to lack of institutional access. |
| Methods | 7 | Present the full search strategies for all databases, registers and websites, including any filters and limits used. | Section 2.2 and Supplementary S2 | Generic search equation provided in main text; full strategies per database in Supplementary Material S2. |
| Methods | 8 | Specify the methods used to decide whether a study met the inclusion criteria of the review. | Section 2.3 | Two-phase screening (title/abstract then full-text) in Rayyan with blind mode. Screening was performed independently by two reviewers (KRJU and NK) in Rayyan with blind mode enabled. Inter-rater agreement: raw agreement 92.4%, Cohen's kappa = 0.84 (almost perfect agreement per Landis and Koch, 1977). Discrepancies (n = 15) were resolved by discussion with consultation of a senior co-author when needed. |
| Methods | 9 | Specify the methods used to collect data from reports. | Section 2.4 | Standardized Excel extraction grid with six worksheets (Identification, Agromorphological, Biochemical, Molecular, Quality, PRISMA summary). |
| Methods | 10a | List and define all outcomes for which data were sought. | Section 2.4 | Diversity metrics: He, Ho, PIC, Na, Fst, Shannon index, CV%; PCA variance; cluster numbers; biochemical components (starch, protein, oxalates); descriptors used. |
| Methods | 10b | List and define all other variables for which data were sought. | Section 2.4 | Bibliographic details, study location (country, region), sample size, marker type, number of markers/descriptors, publication year. |
| Methods | 11 | Specify any processes used to assess risk of bias in the included studies. | Section 2.5 | Ad hoc quality scoring tool inspired by JBI Critical Appraisal Tools. Five common criteria (Q1-Q5) and three type-specific criteria (Q6-Q8). Studies classified as High/Medium/Low quality. |
| Methods | 12 | Specify for each outcome the effect measure(s) used in the synthesis or presentation of results. | Section 2.6.2 | Raw (untransformed) means pooled across studies; effect measure: MRAW for He, PIC, Na. Heterogeneity quantified by I² and Cochran's Q. |
| Methods | 13a | Describe the processes used to decide which studies were eligible for each synthesis. | Section 2.6 | Narrative synthesis for all 64 studies; meta-analysis restricted to 44 original research articles with sufficient quantitative data. |
| Methods | 13b | Describe any methods required to prepare the data for presentation or synthesis. | Section 2.4 | Ranges converted to means (midpoint) with SD approximated as range/4. Standard errors converted to SD via SD = SE × √n. |
| Methods | 13c | Describe any methods used to tabulate or visually display results of individual studies and syntheses. | Section 2.6.2 | Forest plots for pooled estimates; funnel plots for publication bias; subgroup analyses by marker type, region, period; leave-one-out sensitivity plots. |
| Methods | 13d | Describe any methods used to synthesize results and provide a rationale for the choice(s). | Section 2.6.2 | Random-effects model with REML estimator chosen due to expected methodological heterogeneity across studies. Inverse-variance weighting. |
| Methods | 13e | Describe any methods used to explore possible causes of heterogeneity. | Section 2.6.2 | Subgroup analyses by marker type (SSR vs SNP/other), African region (West/East/Southern), and publication period (2010-2015, 2016-2020, 2021-2026). |
| Methods | 13f | Describe any sensitivity analyses conducted to assess robustness of the synthesized results. | Section 2.6.2 | Leave-one-out sensitivity analyses for each outcome; analyses restricted to High-quality studies. |
| Methods | 14 | Describe any methods used to assess risk of bias due to missing results in a synthesis. | Section 2.6.2 | Funnel plots and Egger's regression test for asymmetry (applied when k ≥ 10 studies). |
| Methods | 15 | Describe any methods used to assess certainty (or confidence) in the body of evidence for an outcome. | Section 2.5 and 2.6.2 | Quality assessment via ad hoc scoring tool; certainty discussed through prediction intervals and I² interpretation. |
| **RESULTS** | | | | |
| Results | 16a | Describe the results of the search and selection process, from the number of records identified in the search to the number of studies included in the review, ideally using a flow diagram. | Section 3.1; Figure 1 | 222 identified → 198 screened → 79 full-text → 64 included. PRISMA flow diagram provided as Figure 1. |
| Results | 16b | Cite studies that might appear to meet the inclusion criteria, but which were excluded, and explain why they were excluded. | Section 3.1; Supplementary S3 | 15 full-text exclusions with reasons (no quantitative data, outside Africa, not C. esculenta, duplicate dataset, etc.). Complete list in Supplementary S3. |
| Results | 17 | Cite each included study and present its characteristics. | Section 3.2; Table in Supplementary S3 | 44 original studies + 20 reviews characterized by author, year, country, sample size, marker type, characterization approach. |
| Results | 18 | Present assessments of risk of bias for each included study. | Section 3.3; Table 2 | Quality scores: 32 High, 28 Medium, 4 Low. Distribution presented in Table 2. |
| Results | 19 | For all outcomes, present, for each study: (a) summary statistics for each group and (b) an effect estimate and its precision, ideally using structured tables or plots. | Section 3.7; Figures 2-4 | Individual study estimates of He, PIC, Na with 95% CI presented in forest plots. |
| Results | 20a | For each synthesis, briefly summarise the characteristics and risk of bias among contributing studies. | Sections 3.4-3.6 | Characteristics summarised by characterization type; quality assessment integrated. |
| Results | 20b | Present results of all statistical syntheses conducted. If meta-analysis was done, present for each the summary estimate and its precision and measures of statistical heterogeneity. | Section 3.7; Table 3 | Pooled He = 0.40 [0.32, 0.48], I² = 99.5%; PIC = 0.46 [0.38, 0.55], I² = 92.5%; Na = 3.73 [3.14, 4.32], I² = 89.4%. |
| Results | 20c | Present results of all investigations of possible causes of heterogeneity among study results. | Section 3.7.2 | Subgroup analyses by marker type showed higher He in SSR (0.45) vs other markers (0.31). |
| Results | 20d | Present results of all sensitivity analyses conducted to assess the robustness of the synthesized results. | Section 3.7.6 | Leave-one-out analyses confirmed robustness; pooled He ranged from 0.37 to 0.43 across iterations. |
| Results | 21 | Present assessments of risk of bias due to missing results (arising from reporting biases) for each synthesis assessed. | Section 3.7.5 | Funnel plot for He showed moderate asymmetry; Egger's test non-significant (p = 0.12); interpreted with caution given k = 12. |
| Results | 22 | Present assessments of certainty (or confidence) in the body of evidence for each outcome assessed. | Section 3.7 and Section 4.8 | Certainty discussed through I² values, prediction intervals, and limitations of underlying studies. |
| **DISCUSSION** | | | | |
| Discussion | 23a | Provide a general interpretation of the results in the context of other evidence. | Section 4.1 | Comparison of pooled He with cassava, yam, and other clonally propagated crops. Moderate genetic diversity contextualized within global taro literature. |
| Discussion | 23b | Discuss any limitations of the evidence included in the review. | Section 4.4 and 4.8 | Discussion of methodological heterogeneity, marker-dependent biases, geographic gaps. |
| Discussion | 23c | Discuss any limitations of the review processes used. | Section 4.8 | English-only literature, no Scopus/WoS access, textual data conversion, high heterogeneity, small k for some variables. |
| Discussion | 23d | Discuss implications of the results for practice, policy, and future research. | Section 4.7 and Conclusion | Implications for breeding (high-yielding genotypes), conservation (collection priorities), and research (integrated multi-dimensional studies, SNP genotyping). |
| **OTHER INFORMATION** | | | | |
| Other information | 24a | Provide registration information for the review, including the register name and registration number, or state that the review was not registered. | Sections 2 and Methods | Registered on Open Science Framework (OSF) Registries. Registration DOI: 10.17605/OSF.IO/5S9PY. Date: April 26, 2026. |
| Other information | 24b | Indicate where the review protocol can be accessed, or state that a protocol was not prepared. | Section 2 | Protocol publicly available at https://doi.org/10.17605/OSF.IO/5S9PY |
| Other information | 24c | Describe and explain any amendments to information provided at registration or in the protocol. | Section 2.6 | Original protocol focused only on molecular characterization in West Africa; scope was broadened to include agromorphological and biochemical approaches across all of Africa during early data extraction. Amendment documented on OSF. |
| Other information | 25 | Describe sources of financial or non-financial support for the review, and the role of the funders or sponsors in the review. | Declarations | No specific funding received for this review. Work conducted as part of the ORPHAN Crop Project (EU/MOBAF Project N° 101144314). |
| Other information | 26 | Declare any competing interests of review authors. | Declarations | Authors declare no competing interests. |

Notes on the PRISMA 2020 statement

This checklist follows the PRISMA 2020 statement, which provides updated reporting guidance for systematic reviews. The checklist comprises 27 items, organized across seven sections: Title, Abstract, Introduction, Methods, Results, Discussion, and Other Information.

All items of the PRISMA 2020 checklist were considered during the preparation of this systematic review. Items 16a-16b are reported in the Results section addressing study selection; the PRISMA flow diagram (Figure 1) visually summarises the screening process.

This systematic review was prospectively registered on the Open Science Framework Registries (Registration DOI: 10.17605/OSF.IO/5S9PY) on April 26, 2026, prior to data extraction and synthesis. The complete protocol, data extraction grid, R analysis scripts, and supplementary materials are publicly available through the registered OSF project page.
