# Supplementary material for: Mapping the characterization landscape of Colocasia esculenta (L.) in Africa: gaps, trends, and priorities - a systematic review with meta-analysis
Source: Front Plant Sci. 2026 Sep 21;17:1930197. doi: 10.3389/fpls.2026.1930197 (PMC13635379; doi:10.3389/fpls.2026.1930197)
Supplement: Supplementary file 2 [file SupplementaryFile2.docx]

**Supplementary Material S2**

**Search Strategies per Database**

**Systematic review:** "Mapping the characterization landscape of Colocasia esculenta in Africa: gaps, trends, and priorities from a systematic review with meta-analysis"

**Protocol registration:** OSF Registries (DOI: 10.17605/OSF.IO/5S9PY)

Search period: 1 January 2010 – 30 April 2026

Search dates: 15 March 2026 – 28 April 2026

Language restriction: English

Search performed by: Riwan Jaurès Uriel KOUKE. Screening performed independently by KRJU and NK (Cohen's kappa = 0.84).

**1. Overview of databases searched**

Five electronic databases were searched to identify potentially eligible studies. The selection of databases aimed to maximize coverage of both international and African-specific literature on *Colocasia esculenta* characterization. Scopus and Web of Science were not used due to lack of institutional access at the corresponding author's institution; OpenAlex was included as a complementary open-access alternative providing broad indexing coverage.

| **Database** | **URL / Access** | **Records retrieved** |
| --- | --- | --- |
| PubMed | <https://pubmed.ncbi.nlm.nih.gov/> | 17 |
| Google Scholar | <https://scholar.google.com/> | 152 |
| AGRIS (FAO) | <https://agris.fao.org/> | 2 |
| AJOL | <https://www.ajol.info/> | 25 |
| OpenAlex | <https://openalex.org/> | 26 |

**2. Generic search equation**

The following generic search equation was developed and adapted to the specific syntax and search capabilities of each database. Terms within each conceptual block were combined with the Boolean operator OR; the three blocks were combined with the Boolean operator AND.

**Block 1 — Population (taro / *Colocasia esculenta*) :**

"Colocasia esculenta" OR taro OR cocoyam OR dasheen OR eddoe

**Block 2 — Characterization approaches:**

characterization OR characterisation OR diversity OR genetic OR morphological OR agromorphological OR phenotypic OR biochemical OR nutritional OR proximate OR molecular OR SSR OR microsatellite OR SNP OR AFLP OR RAPD OR ISSR OR GBS

**Block 3 — Geographic scope (Africa):**

Africa OR "West Africa" OR "East Africa" OR "Southern Africa" OR "Central Africa" OR Nigeria OR Ghana OR Ethiopia OR Kenya OR Cameroon OR "South Africa" OR "Côte d'Ivoire" OR "Ivory Coast" OR Tanzania OR Uganda OR Malawi OR Togo OR Benin OR "Burkina Faso" OR Senegal OR Mali OR Congo OR Madagascar

**3. Database-specific search strategies**

**3.1 PubMed (NCBI)**

Date of search: 15 March 2026

Filters applied: Publication date from 2010/01/01 to 2026/04/30; English language

Records retrieved: 17

**Search query:**

(("Colocasia esculenta"[Title/Abstract] OR taro [Title/Abstract] OR cocoyam [Title/Abstract] OR dasheen [Title/Abstract] OR eddoe [Title/Abstract])

AND

(characterization [Title/Abstract] OR characterisation[Title/Abstract] OR diversity [Title/Abstract] OR genetic [Title/Abstract] OR morphological [Title/Abstract] OR agromorphological[Title/Abstract] OR biochemical [Title/Abstract] OR nutritional [Title/Abstract] OR molecular [Title/Abstract] OR SSR[Title/Abstract] OR microsatellite [Title/Abstract] OR SNP[Title/Abstract] OR AFLP[Title/Abstract] OR RAPD[Title/Abstract] OR ISSR[Title/Abstract])

AND

(Africa [Title/Abstract] OR "sub-Saharan Africa"[Title/Abstract] OR Nigeria [Title/Abstract] OR Ghana [Title/Abstract] OR Ethiopia [Title/Abstract] OR Kenya [Title/Abstract] OR Cameroon [Title/Abstract] OR "South Africa"[Title/Abstract] OR "Côte d'Ivoire"[Title/Abstract] OR Tanzania [Title/Abstract] OR Uganda [Title/Abstract] OR Malawi [Title/Abstract] OR Togo [Title/Abstract]))

AND ("2010/01/01"[Date - Publication]: "2026/04/30"[Date - Publication])

AND English [Language]

Notes: Three independent searches were performed by varying the characterization keyword and combining results. Duplicates between searches were removed.

**3.2 Google Scholar**

Date of search: 22 March 2026

Filters applied: Custom range 2010–2026; English language; "Articles" only

Records retrieved: 152 (first 200 results examined per search)

**Search query (main):**

"Colocasia esculenta" OR taro AND (characterization OR diversity OR molecular OR SSR OR SNP) AND (Africa OR Nigeria OR Ghana OR Ethiopia OR Kenya OR Cameroon)

**Additional searches:**

"Colocasia esculenta" "Africa" agromorphological characterization

"Colocasia esculenta" "Africa" biochemical nutritional composition

taro "Africa" SSR microsatellite genetic diversity

Notes: Google Scholar limits complex Boolean queries. Four separate searches were performed using progressively narrower keyword combinations. The first 200 results were examined for each search (sorted by relevance), and unique titles meeting the inclusion criteria were exported to Zotero. Google Scholar's lack of advanced filtering required manual screening of the publication year for each result.

**3.3 AGRIS (FAO International System for Agricultural Science and Technology)**

Date of search: 5 April 2026

Filters applied: Date range 2010–2026; English language; document type: Journal article

Records retrieved: 2

**Search queries:**

"Colocasia esculenta" AND Africa

taro AND characterization AND Africa

taro AND diversity AND Nigeria

taro AND molecular AND Ethiopia

cocoyam AND characterization AND Africa

"Colocasia esculenta" AND nutritional AND Africa

Notes: AGRIS has limited Boolean operator support compared to PubMed. Six separate searches were performed with simpler keyword combinations covering each characterization approach and major taro-producing African countries. Despite the database's focus on agricultural literature, very few records on Colocasia esculenta from Africa were indexed, reflecting the limited representation of African taro research in this database.

**3.4 AJOL (African Journals Online)**

Date of search: 12 April 2026

Filters applied: All disciplines; English language; date range 2010–2026

Records retrieved: 25

**Search queries:**

"Colocasia esculenta"

taro AND characterization

taro AND diversity

cocoyam AND Africa

"Colocasia esculenta" AND molecular

Notes: AJOL is the largest collection of peer-reviewed African journals. The platform supports simple keyword searches but limited Boolean operations. Five separate searches were performed to maximize coverage. AJOL was particularly valuable for identifying studies published in African regional journals such as the African Journal of Biotechnology, the African Journal of Agricultural Research, and similar publications that are often indexed in international databases with lower coverage.

**3.5 OpenAlex**

Date of search: 28 April 2026

Filters applied: Publication year 2010–2026; language: English; type: journal-article

Records retrieved: 26

**Search queries:**

"Colocasia esculenta" AND Africa AND (characterization OR diversity)

taro AND ("genetic diversity" OR "molecular characterization") AND Africa

"Colocasia esculenta" AND ("SSR" OR "SNP" OR "RAPD" OR "AFLP") AND Africa

taro AND ("agromorphological" OR "biochemical") AND Africa

Notes: OpenAlex is an open and comprehensive index of scholarly works, replacing the discontinued Microsoft Academic Graph. The OpenAlex web interface (https://openalex.org/) was used for the searches, with the advanced query builder. Four separate searches covering each characterization approach were performed.

**4. Deduplication and screening process**

All retrieved records from the five databases were exported in RIS format and imported into Zotero (version 6.0) for initial deduplication. Zotero's built-in duplicate detection algorithm was used, supplemented by manual verification of borderline cases. The deduplicated set was then imported into Rayyan (<https://www.rayyan.ai> ) for systematic screening. Rayyan's automated deduplication function identified additional duplicates that had been missed by Zotero, particularly cases involving preprint and published versions of the same article.

**Total references identified: 222**

**Duplicates removed: 24 (Rayyan: 14; Zotero: 10)**

**Unique references screened: 198**

**5. Limitations of the search strategy**

The following limitations of the search strategy are acknowledged and discussed in the main manuscript (Section 4.8):

First, the search was restricted to English-language publications, which may have excluded relevant studies published in French, Portuguese, or other languages spoken in taro-growing African countries. This is particularly relevant for francophone countries such as Côte d'Ivoire, Cameroon, Benin, Burkina Faso, Togo, and Madagascar.

Second, Scopus and Web of Science were not accessible due to lack of institutional subscription at the corresponding author's institution. While OpenAlex was used as an open-access alternative providing broad indexing coverage, some studies indexed exclusively in Scopus or Web of Science may have been missed.

Third, conference proceedings, theses, and other grey literature were not systematically searched. African universities produce substantial postgraduate research on taro that may not be indexed in the databases consulted. ProQuest Dissertations & Theses Global was not accessible.

**6. Search update**

No search update was performed before manuscript submission. The original search of 28 April 2026 represents the final search date. A search update is recommended at the time of manuscript revision if the time elapsed between the original search and submission exceeds 12 months.
