# Supplementary material for: Mapping the characterization landscape of Colocasia esculenta (L.) in Africa: gaps, trends, and priorities - a systematic review with meta-analysis
Source: Front Plant Sci. 2026 Sep 21;17:1930197. doi: 10.3389/fpls.2026.1930197 (PMC13635379; doi:10.3389/fpls.2026.1930197)
Supplement: Supplementary file 3 [file SupplementaryFile3.docx]

**Supplementary Material S3**

**Characteristics of Included Studies**

Systematic review: "Mapping the characterization landscape of *Colocasia esculenta* in Africa: gaps, trends, and priorities from a systematic review with meta-analysis"

This table presents the characteristics of all 44 original research articles included in the systematic review and quantitative meta-analysis. Studies are sorted chronologically by year of publication, then alphabetically by first author surname. Review articles (n = 20) included in the narrative synthesis are presented separately in Table S3.2.

*Abbreviations: ID = Study identifier; Acc. = Number of accessions evaluated; Char. = Characterization type (Agro = Agromorphological, Bio = Biochemical, Mol = Molecular, Agro/Mol = combined agromorphological and molecular); Quality = Methodological quality assessment based on ad hoc scoring tool (High ≥ 8, Medium 5–7, Low < 5); NR = Not reported.*

**Table 1**: Characteristics of the 44 original research articles included in the meta-analysis

| ID | Author(s) & Year | Country | Region | Acc. | Characterization | Quality |
| --- | --- | --- | --- | --- | --- | --- |
| S010 | Greyling et al. (2011) | South Africa | South | 92 | Mol | High |
| S061 | Beyene & co. (2013) | Ethiopia | East | 100 | Agro | High |
| S023 | Mulualem et al. (2013) | Ethiopia | East | 100 | Agro | High |
| S060 | Rensburg et al. (2013) | South Africa | South | 92 | Mol | High |
| S063 | Rensburg et al. (2013) | South Africa | South | 92 | Mol | High |
| S001 | Macharia et al. (2014) | Kenya, Tanzania, Uganda | East | 98 | Mol | High |
| S007 | Nguluta & co. (2014) | South African | South | NR | Bio | High |
| S051 | Osawaru et al. (2014) | Nigeria | West | 54 | Agro | High |
| S031 | Akwee et al. (2015) | Kenya and Pacific Islands | NR | 50 | Agro | High |
| S030 | Ezeabara et al. (2015) | Nigeria | West | 5 | Agro | Low |
| S022 | Osawaru et al. (2015) | Nogeria | West | 36 | Mol | High |
| S016 | Osawaru et al. (2015) | Nigeria | West | 36 | Mol | High |
| S004 | Chaïr et al. (2016) | Asia, Africa, America, Madeira, Pac | Asia, Africa, America, Madeira | 321 | Mol | High |
| S039 | Eze & co. (2016) | Nigeria | West | 8 | Agro | Low |
| S021 | Mwenye et al. (2016) | Malawi | South | 28 | Agro/Mol | High |
| S005 | Nguluta et al. (2016) | South Africa | South Africa | 25 | Mol | High |
| S002 | Palapala et al. (2016) | Kenya | East | 25 | Mol | High |
| S057 | Palapala et al. (2016) | Kenya | East | 50 | Mol | High |
| S015 | Edome & co. (2017) | Kenya | East | 24 | Mol | Medium |
| S046 | Gerrano et al. (2017) | South Africa | South | 29 | Agro | Medium |
| S055 | Muinat et al. (2017) | South Africa | South | 6 | Agro | Medium |
| S047 | Peter Edome et al. (2017) | Kenya | East | 50 | Agro | High |
| S043 | Boampong et al. (2018) | Ghana | West | 36 | Agro | High |
| S019 | Gerrano et al. (2018) | South Africa | South | 29 | Agro | Medium |
| S017 | Grimaldi et al. (2018) | Nigeria, Cameroon, the Democratic R | NR | 20 | Agro | High |
| S006 | Bammite et al. (2021) | Togo | West | 26 | Mol | High |
| S041 | Fufa et al. (2021) | Nigeria | West | 25 | Agro | Medium |
| S050 | Gerrano et al. (2021) | South Africa | South | 14 | Bio | Medium |
| S033 | Koffi et al. (2021) | Côte d'Ivoire | West | 213 | Agro | High |
| S003 | Fufa et al. (2022) | Nigeria and Vanuatu | West | 282 | Mol | High |
| S045 | Akwee et al. (2023) | Kenya | East | 25 | Agro | Medium |
| S011 | Fufa et al. (2023) | Nigeria | West | 92 | Mol | High |
| S038 | Fufa et al. (2023) | Nigeria | West | 188 | Bio | High |
| S024 | Fufa et al. (2023) | Nigeria | West | 100 | Agro | High |
| S062 | Ouédraogo et al. (2023) | Burkina Faso | West | 59 | Bio | High |
| S053 | Alfred et al. (2024) | Nigeria | West | 5 | Agro | Low |
| S008 | Amoako et al. (2024) | Ghana | West | 26 | Mol | High |
| S064 | Banti et al. (2024) | Ethiopia | East | 6 | Bio | Low |
| S036 | Oladimeji et al. (2024) | Nigeria | West | 459 | Agro/Mol | High |
| S013 | Sinkiyian Kepue & co. (2024) | Kenya | East | 186 | Mol | High |
| S049 | Debela et al. (2025) | Ethiopia | East | 64 | Agro | High |
| S014 | Emmanuel et al. (2025) | Nigeria | West | 20 | Mol | Medium |
| S020 | Oladimeji & co. (2025) | Nigeria | West | 490 | Agro/Mol | High |
| S009 | Alelign et al. (2026) | Nigeria | West | 25 | Mol | High |

**Table S3.2. Review articles included in the narrative synthesis**

Twenty review articles, editorials, and synthesis papers were identified during screening. These articles did not provide primary research data on Colocasia esculenta characterization in Africa and were therefore excluded from the quantitative meta-analysis. However, they provided important contextual information, identified knowledge gaps, and proposed research priorities that were integrated into the narrative discussion. These review articles are listed below for completeness.

Note: The complete reference list for all 64 included studies (original research and reviews) is provided in the main manuscript's reference list and in Supplementary Material S5 (data extraction grid).

**Notes on data extraction**

Data extraction was performed by a single reviewer using a standardized Excel grid with six worksheets covering identification, agromorphological data, biochemical data, molecular data, quality assessment, and PRISMA summary. The complete data extraction grid is provided as Supplementary Material S5.

When quantitative data were reported as ranges rather than means with standard deviations, the midpoint of the range was used as the mean estimate, and the range divided by four was used as an approximate standard deviation following the recommendations of Hozo et al. (2005). When standard errors were reported, they were converted to standard deviations using the formula SD = SE × √n, where n is the number of loci or accessions analysed.

Studies reporting characterization data on multiple geographic populations within the same publication were treated as a single entry in the database, with country and region information reflecting the broadest geographic scope of the study.
