# Supplementary material for: Mapping the characterization landscape of Colocasia esculenta (L.) in Africa: gaps, trends, and priorities - a systematic review with meta-analysis
Source: Front Plant Sci. 2026 Sep 21;17:1930197. doi: 10.3389/fpls.2026.1930197 (PMC13635379; doi:10.3389/fpls.2026.1930197)
Supplement: Supplementary file 4 [file SupplementaryFile4.docx]

Supplementary Material S4

Descriptive Summary of Biochemical Parameters

Systematic review: "Mapping the characterization landscape of *Colocasia esculenta* in Africa: gaps, trends, and priorities from a systematic review with meta-analysis"

Overview

This supplementary table provides a descriptive summary of the biochemical parameters reported across the biochemical characterization studies of African *Colocasia esculenta* included in this systematic review. Given the methodological heterogeneity between studies, a formal quantitative meta-analysis was not appropriate. This descriptive summary is intended as a reference tool for researchers designing new biochemical characterization studies of taro.

Supplementary Table S4. Descriptive summary of biochemical parameters reported across four studies characterising African Colocasia esculenta accessions.

| **Parameter** | **Unit** | **Range (min-max)** | **Mean** | **N total** | **k** | **CV (%)** | **Studies** |
| --- | --- | --- | --- | --- | --- | --- | --- |
| **PROXIMATE COMPOSITION** |  |  |  |  |  |  |  |
| Crude protein (Kjeldahl/AOAC) | % dw | 3.38 - 9.36 | 7.22 | 194 | 2 | 11.1 | S003, S004 |
| Crude lipids | % dw | 0.05 - 1.23 | 0.65 | 194 | 2 | 10.9 | S003, S004 |
| Crude fibre | % dw | 1.40 - 5.06 | 2.88 | 194 | 2 | 55.9 | S003, S004 |
| Ash | % dw | 0.09 - 7.14 | 4.34 | 194 | 2 | 61.3 | S003, S004 |
| Starch (enzymatic) | % dw | 0.31 - 17.56 | 12.96 | 59 | 1 | 28.2† | S001 |
| Moisture / Dry matter | % | 5.25 - 30.00 | — | 194 | 2 | — | S003, S004 |
| **MINERAL COMPOSITION** |  |  |  |  |  |  |  |
| Iron (Fe) | mg/100g dw | 2.20 - 56.70 | 22.6 | 208 | 3 | 65.4 | S002, S003, S004 |
| Zinc (Zn) | mg/100g dw | 0.15 - 120.00 | — | 208 | 3 | high | S002, S003, S004 |
| Copper (Cu) | mg/100g dw | 2.16 - 4.54 | 3.35 | 14 | 1 | — | S002 |
| Calcium (Ca) | mg/100g dw | 7.31 - 176.10 | — | 208 | 3 | high | S002, S003, S004 |
| Magnesium (Mg) | mg/100g dw | 17.00 - 62.00 | 39.5 | 6 | 1 | 40.3 | S003 |
| Potassium (K) | mg/100g dw | 476 - 2525 | ~1500 | 194 | 2 | 35.4 | S003, S004 |
| **ANTI-NUTRITIONAL FACTORS** |  |  |  |  |  |  |  |
| Total oxalates | mg/100g dw | 29.6 - 440.1 | 234.9 | 188 | 1 | high† | S004 |
| Phytates | mg/100g dw | 6.28 - 28.90 | 17.59 | 6 | 1 | 64.4† | S003 |
| Condensed tannins | mg/100g dw | 57.40 - 134.00 | 95.7 | 6 | 1 | 40.0† | S003 |
| **BIOACTIVE / FUNCTIONAL COMPOUNDS** |  |  |  |  |  |  |  |
| Total phenolics | mg GAE/100g dw | 4.37 - 115.21 | 59.79 | 59 | 1 | 115.9† | S001 |
| Flavonoids | mg QE/100g dw | 0.25 - 1.47 | 0.86 | 59 | 1 | high† | S001 |

*Notes: All values expressed on a dry-weight basis (dw). Range = minimum-maximum values reported across studies; Mean = arithmetic mean of study-level means (rounded to two decimals); N total = total number of accessions across all studies reporting the parameter; k = number of studies reporting the parameter; CV (%) = coefficient of variation calculated as (standard deviation of study-level means / mean of means) x 100. Values marked with † represent within-study CVs where only one study reported the parameter or where within-study heterogeneity dominates. Where mean of means could not be meaningfully computed due to methodological heterogeneity, values are shown as "—". Included studies: S001 (n = 59 accessions, focused on phenolics and functional compounds), S002 (n = 14, focused on mineral composition), S003 (n = 6, complete nutritional profile), S004 (n = 188, complete nutritional profile with detailed oxalate speciation).*
